# Experiences of Social Media Platforms’ Policies and Restrictions Related to Self-Harm Content: Mixed Methods Study

**DOI:** 10.2196/73343

**Published:** 2026-08-06

**Authors:** Amanda Marchant, Fran Lewis, Moiz Siddiqi, Ann John

**Affiliations:** 1 National Centre for Suicide Prevention and Self-harm Research, Swansea University, 3rd Floor, Data Science Building, Swansea, SA2 8PP, United Kingdom, 44 0 1792 60

**Keywords:** self-harm, social media, survey, in-depth interviews, suicide

## Abstract

**Background:**

Many features of social media platforms can influence safety in relation to self-harm/suicide-related content. In 2019, platforms including Meta updated their policies regarding self-harm/suicide content. This included community guidelines, content restrictions, and signposting. The impact of these safety measures has yet to be fully evaluated.

**Objective:**

This study aimed to better understand the perspectives of social media users on safety measures related to self-harm/suicide content, including perspectives of those with a history of self-harm.

**Methods:**

This was a cross-sectional mixed methods study. Participants were recruited through convenience sampling. Quantitative data were collected using a fixed-response cross-sectional survey (5294 respondents aged 16-84 years, largely women/girls or nonbinary). Questions related to the experience of self-harm and suicide content, the change policy in 2019, and specific features of platforms. Qualitative data were collected via semistructured interviews (17 participants aged 16‐56 years) with interview guides broadly following the topic areas of the survey. Interviews were analyzed using thematic analysis.

**Results:**

Half of the survey respondents (49.8%, 95% CI 47.8%-51.8%; 2476/4970) stated the current restrictions made social media feel safer, 34.9% (95% CI 32.6%-37.2%; 1736/4981) reported that this changed what they saw on social media, and just 9.9% (95% CI 7.5%‐13.0%; 492/4479) stated that this changed what they posted. Furthermore, 64.3% (95% CI 62.6%-65.9%; 3212/4997) stated that they would be “very likely” to click on a post marked with a generic content warning, with 15.1% (95% CI 12.6%‐17.9%; 753/4998) being “very likely” to click on a post with a self-harm specific content warning. Of the respondents who had content removed due to mental health or self-harm content, ~90% indicated that this was harmful or very harmful to them at the time, particularly where content was removed because of visible scars (extremely harmful 58.3%, 95% CI 54.3%‐62.3%; 599/1027; harmful 31.3%, 95% CI 26.3%‐36.7%; 321/1027). Thematic analysis of in-depth interviews revealed 13 subthemes organized into 7 overarching thematic areas. Participants described the experience of social media platforms before and after the policy changes, with opinions divided on whether existing safety features were effective. Participants described the distress caused by inappropriate censoring of images, for example, where healed self-harm scars were visible. Suggestions were made for how platforms can continue to improve, including age verification procedures for young people, tailored signposting, and increased control over content.

**Conclusions:**

To our knowledge, this is the first study to explore the 2019 change in platform policies with respect to self-harm/suicide-related content from the perspectives of those with a history of self-harm. This research adds to the evidence of the risks and unintended consequences of imposing untested blanket restrictions in online settings. Full evaluation of these restrictions is essential to allow platforms to continue to improve, encouraging a safe space for supportive communities while mitigating potential harm.

## Introduction

Young people who self-harm spend more time online than others of a similar age [1]. There is a large volume of self-harm–related material online [2]. Young people, policymakers, and social media industry professionals highlight the use of social media as a space to seek and provide support and to share and understand personal experiences of self-harm and suicide in conjunction with concerns related to the safety of conversations, particularly for younger adolescents [3]. Literature reviews demonstrate both harmful and protective aspects of digital spaces in relation to self-harm and suicide content. Positive aspects include opportunities to access information, community and crisis support, and as a creative outlet; however, this exists alongside the potential for harm with opportunities to access content that normalizes, encourages, or promotes self-harm and suicide [2,4,5].

There are many features of social media platforms that can influence safety. Easy searching of images, perceived anonymity, and a lack of moderation may contribute to potentially harmful online environments [4]. Content or “trigger warnings” are designed to warn people about potentially distressing or graphic content. These are widely used in online spaces; however, there is debate about their effectiveness in reducing distress. A recent meta-analysis concluded that trigger warnings rarely result in avoidance of content and have no effect on emotional reactions [6]. Current safety tools have been described as limited, with the need for better collaboration across sectors [3].

There have been recent calls to ensure that policies and restrictions are evidence based and do not inadvertently lead to harm, shame, and increased isolation [7]. Blanket bans of self-harm images may leave some individuals without a support system that they previously leaned on [8], and risk impeding pathways for help-seeking [9]. Safety concerns need to be balanced with the potential benefits, and issues of censorship and stigma should not be overlooked in any restrictions [10]. A range of recommendations for social media platform safety policy were identified in a recent review, including tighter legislation for platforms and enabling greater autonomy and control over content for platform users [11]. However, a lack of evidence-based recommendations was noted [11].

Additional safety features in relation to self-harm and suicide content were introduced by many social media platforms in 2019 (eg, [12]). These include community guidelines, blurring or removal of images, tools for reporting content, messaging related to self-harm, signposting, and content warnings, among others. While the features and messaging introduced by platforms have been put in place to improve safety, they have not been fully evaluated. Previous research has explored changes in content on Twitter (now “X”) following these changes [13]. However, to our knowledge, research has yet to gain perspectives of individuals with a history of self-harm.

The aim of this study was to use a mixed methods approach to better understand the perspectives of social media users, including those with a history of self-harm. Through a survey and in-depth interviews, we collected data on experiences of current policies and safety features, including censoring or removal of posts, policies for reporting of posts, help messaging, and signposting.

## Methods

### Study Design and Setting

This was a mixed methods study consisting of a cross-sectional survey and semistructured in-depth interviews. The CHERRIES (Checklist for Reporting Results of Internet E-Surveys) [14] and the JARS-Mixed (APA Journal Article Reporting Standards for Mixed Methods Research) [15] checklists were followed.

### Participants

Participants aged 16 years and older (with no upper age limit), with and without a history of self-harm, were recruited. Self-harm refers to any intentional act of self-injury or poisoning independent of motivation or suicidal intent [16]. Both individuals with and without a history of self-harm may see this content on social media; as such, we aimed to capture experiences of both groups.

### Recruitment

Participants for both the survey and in-depth interviews were recruited through convenience sampling. Recruitment was facilitated by the Self-Harm Research UK (SHARE UK) research register, part of the National Centre for Suicide Prevention and Self-Harm Research (NCSR) at Swansea University (Adolescent Mental Health Data Platform [ADP] [17]). This is a register of more than 2000 individuals who have consented to be contacted to take part in research studies related to self-harm. Sign-up to SHARE UK is open to anyone aged 16 years and older who resides in the United Kingdom (including those with and without a history of self-harm). Participants were also recruited via two social media campaigns, one specific to the survey and one specific to the interviews. Survey responses were anonymous. It was not possible to measure whether individuals took part in just the survey/in-depth interviews or both.

### Patient and Participant Involvement

Three online focus groups were conducted between January 17, 2022, and January 20, 2022. The purpose of these focus groups was to inform the topics covered in the survey and in-depth interviews. Further details of the focus groups' design, analysis, and themes can be found in Multimedia Appendix 1. Analysis of focus group data revealed 6 thematic areas to be further developed in the survey and in-depth interviews (Table 1). The survey was piloted and refined following feedback from individuals from the SHARE UK research register, and in-depth interview schedules were designed to mirror the topics covered.

**Table 1.** Themes for survey and interviews identified during coproduction stages.

Theme	Description
Experiences of viewing self-harm/suicide-related content	With subtopics to include the age when this content was viewed, which platforms, whether this content was searched or suggested, and the impact on the person’s mood or self-harm. We also explored experiences of having or following dedicated self-harm or recovery accounts.
Current policies and restrictions related to self-harm/suicide content	These included experiences of additional features (eg, censoring, restrictions on searches) being introduced in 2019. Not all participants reported noticing a change in what they viewed or shared as a result of this change. Many talked about an absence of clear messaging around the change in policy. Individuals who used these accounts for journaling expressed sadness when their content was removed. Participants talked at length about the possibility for restrictions to leave people feeling isolated and less likely to open up in the future. There were mixed responses as to whether these features made social media safer.
Censoring, blurring, or removal of self-harm content	This includes how this has affected the content people see, whether this is effective in preventing people from viewing graphic posts, and the experiences of having images censored. We explored this in relation to self-harm content; images of healed self-harm scars and posts related to positive mental health and recovery. Focus group participants discussed the potential for censoring to draw more attention to the person who posted the original images. Examples were given of prom pictures where self-harm scars were visible being marked with a warning.
Procedures when posts are reported	This includes experiences both for the person who reported the post and for the individual who posted it. We also explored what happens if a post is reported for concerns over a person’s well-being or safety.
Help messaging and signposting	Signposting to sources of help was frequently discussed. It was highlighted that not all signposting was made equal. Suggestions for how this feature could be improved were discussed.
Possible changes to platform features	Suggestions for changes to current policies included increased moderation, changes to targeted advertising, features for people to generate their own specific content/trigger warnings, and easy-to-mute keywords/hashtags.

### Measures and Outputs

#### Survey

The survey consisted of 55 questions split into the following sections:

- Participant information: This included age, gender identity, ethnicity, and history of self-harm.
- Experience of self-harm and suicide content on social media platforms: This included questions on platforms used and the impact on mood and self-harm.

- Change in policy in 2019: This section asked respondents whether they felt that the changes to policy and additional safety features changed what they posted or viewed on social media, and whether they made social media safer.

- Specific safety features: This section related to the experience of specific features of platforms, including censoring or blocking of posts, content/trigger warnings, signposting and help messaging, reporting of posts, and suspension of accounts.

A full copy of all questions with responses is included in Multimedia Appendix 2.

#### In-Depth Interviews

Interviews followed a semistructured format with interview guides broadly following the format of the fixed-response survey above (interview schedule available on request).

### Data Collection

Survey data were collected online between March 28, 2022, and June 6, 2022. The survey was hosted on SurveyMonkey (including participant information, consent, and debrief forms) due to its user-friendly interface, capability to record anonymous responses, and incorporation of bot detection tools including reCAPTCHA and in-email bot detection to ensure genuine responses.

In-depth interviews were conducted between April 25, 2022, and May 17, 2022. Interviews were conducted one-on-one between a researcher and participant, with a clinician on hand if needed. Interviews were conducted via a messaging app. This format has been previously used to examine perspectives of posting self-harm–related content on social media [18]. The use of messaging apps provides an accessible format that participants are comfortable using. In-depth interviews followed a semistructured format and lasted around 1 hour.

### Data Analysis

#### Survey Data

Survey participants were required to answer a minimum of the demographic information, self-harm history, and at least two further closed-ended questions to be included in the analysis. Participants who had never viewed self-harm material online or who did not meet the minimum data requirement were excluded. Participants were not stratified into those who had or had not experienced self-harm due to low numbers in the no self-harm group.

Due to the exploratory nature of this study and use of a convenience sample, results were not intended to be generalizable to the wider population, and as such, analysis was largely descriptive. Descriptive statistics were used to summarize group characteristics and proportions of people across closed-end questions (counts, percentages, and 95% CIs). Wilson score with continuity correction was used to calculate CIs [19]. Due to the sample being predominantly young people aged 16‐24 years with <5% men/boys and nonrandom item noncompletion, stratification by age and gender would not have been meaningful.

We investigated the number of nonresponses by age, gender, and history of self-harm by chi-square tests on the response rates on each factor after cross-tabulations for each question. As this study is exploratory, analyses were conducted using observed responses only. For each item, percentages were calculated using the number of respondents providing a valid response, with the number missing reported.

Statistical analyses were performed with SPSS version 26.

#### In-Depth Interviews

Transcripts of in-depth interviews were downloaded with participant consent. Each individual was given a participant number to replace their name, and any names or identifiable information present in transcripts was masked. Completed transcripts were reviewed to ensure participant anonymity was retained.

Thematic analysis was used to identify shared patterns of meaning across interviews [20]. This analysis was guided by an interpretive approach, recognizing that participants’ accounts reflect how they understand and make sense of their experiences. Data were sorted thematically, using a code-and-retrieve system [21]. A coding frame was developed through a process of reading and rereading interviews and inductive generation of codes and repeated discussions (AM, AJ, and MS). Reflexivity was addressed through repeated analytic discussions within the research team, during which alternative interpretations and assumptions were critically examined. The coding frame was further informed by the themes developed during the focus groups and previous literature [2,4,5,22-29]. All interviews were coded by one member of the study team (AM) and cross-checked by a senior member of the team (AJ). Themes were organized using NVivo 12 (Lumivero).

### Integration of Quantitative and Qualitative Data

A convergent mixed methods design was used. The survey and in-depth interviews were coproduced with lived experience experts, with the topics in the interviews designed to mirror those covered in the survey. This provided a shared foundation for both quantitative and qualitative data, with alignment between survey items and interview topics facilitating comparison. Survey and interview data were collected and analyzed independently and integrated at the interpretation stage. Quantitative findings were used to describe the distribution of experiences (breadth), while qualitative data from in-depth interviews provided in-depth insight into how participants understood and interpreted these experiences (depth), with this data used to contextualize and expand upon survey findings.

### Ethical Considerations

Ethics approval was granted from the Swansea University Medical School’s Ethics Committee (approval number 2021‐0068). Adherence to Samaritans’ Research Ethics Policy was ensured. Written informed consent was collected for both the survey and in-depth interviews prior to participation. Participants were informed of their right to withdraw. Informed consent was collected with a form hosted on SurveyMonkey containing detailed study information and contact details of the research team (copy of form available on request). Identifiable data were removed prior to analysis of data (see above). No identifiable images were collected as part of this study. Individuals who took part in the in-depth interviews were reimbursed with a £25 (£1=US $1.34 as of July 2026) voucher. No incentives were offered for survey completion. Protocols were developed to identify distress and provide appropriate support. In-depth interviews were conducted by a senior researcher (AM) with a clinical academic present (AJ).

## Results

### Survey

#### Overview

Of the 6801 survey respondents, 5294 met the criteria for inclusion in data analysis. Individuals were aged 16‐84 years (average age 18.9 years). Of the 5294 participants, 95.1% (n=5036) had a history of self-harm. Overall, most participants were aged 16‐24 years and identified as woman/girl or nonbinary (Table 2).

Nonresponse rates for each question ranged from 0.1% to 31.9%. Nonresponse rates were not random. Individuals with genders other than man/boy or woman/girl, those with a history of self-harm, and individuals aged younger than 35 years were less likely to skip individual items (Multimedia Appendix 3). Percentages reflect the number of people who answered each item with missing data omitted. A summary of responses to all survey questions is shown in Multimedia Appendix 2.

**Table 2.** Demographics (age, gender identity, sex, and ethnicity) of survey respondents.

	Survey participants (n=5294), n (%)	Interview participants (n=17), n (%)
Age group (years)		
16‐17	3215 (60.7)	2 (11.8)
18‐24	1614 (30.5)	9 (52.9)
25‐34	327 (6.3)	5 (29.4)
35+	138 (2.6)	1 (5.9)
Gender identity		
Woman/girl	3505 (66.2)	8 (47.1)
Man/boy	234 (4.4)	5 (29.4)
Transman/transwoman	394 (7.4)	0 (0.0)
Nonbinary/genderqueer/agender/gender fluid	880 (16.6)	3 (17.6)
Other/don’t know/PNA^a^	281 (5.3)	1 (5.9)
Sex assigned at birth		
Female	4933 (93.2)	11 (64.7)
Male	218 (4.1)	5 (29.4)
Other/PNA	7 (2.7)	1 (5.9)
Ethnicity		
White/White British	4360 (73.6)	11 (64.7)
Asian/Asian British	189 (3.2)	1 (5.9)
Black/Black British	89 (1.5)	4 (23.5)
Mixed/multiple ethnic groups	865 (14.6)	0 (0.0)
Other/unknown	421 (7.1)	1 (5.9)

a PNA: prefer not to answer.

#### Experiences of Viewing Self-Harm/Suicide Related Content

Viewing self-harm content was reported to be either always or often accidental by 43.2% (95% CI 41.2%‐45.3%; 1405/5283) of respondents (often intentional 19.9%, 95% CI 17.5%‐22.5%; 1051/5283; always/mostly intentional 8.2%, 95% CI 5.9%‐11.3%; 433/5283; unsure/neither accidental nor intentional 28.6%, 95% CI 26.3%‐31%; 1511/5283). Furthermore, 82.9% (95% CI 81.7%‐84%; 4376/5280) had viewed self-harm content that they did not search for. A total of 68.8% (95% CI 68.3%‐70.3%; 3639/5290) of survey respondents were aged 11‐14 years, 17.4% (95% CI 16.5%‐20.1%; 921/5290) were aged 10 years or younger, and just 4.5% (95% CI 3.4%‐8.2%; 236/5290) were aged 17 years or older the first time they viewed self-harm content on social media.

When asked about the impact of seeing or sharing self-harm content online, 34.9% (95% CI 32.7%‐37.1%; 1844/5282) of survey respondents reported a worsening of mood, with only 1.5% (95% CI 0.1%‐8.1%; 80/5282) reporting that this content improves their mood. For over half (52.8%, 95% CI 50.9%-54.6%; 2788/5282), the impact depended on their emotions at the time. Of those with a history of self-harm, more than three-quarters of participants (76.8%, 95% CI 75.4%‐78.1%; 3806/5021) reported self-harming more severely as a result of seeing self-harm content online, with similar proportions having performed the same or very similar self-harm (75.8%, 95% CI 74.4%‐77.1%; 3858/5021). More than a third (39.2%, 95% CI 37.1%‐41.3%; 2065/5273) of respondents had maintained an account which is separate from their personal account dedicated to mental health or self-harm recovery.

#### Change in Policy in 2019

We asked participants about the change in policy restrictions in 2019. Around a third (34.9%, 95% CI 32.6%‐37.2%; 1736/4981) reported that this changed what they saw on social media, and just 9.9% (95% CI 7.5%‐13%; 492/4479) stated that this changed what they posted. In response to the question “Do you think these restrictions make social media safer?” 49.8% (95% CI 47.8%‐51.8%; 2476/4970) answered yes and 50.2% (95% CI 48.2%‐52.2%; 2494/4970) answered no. More than three-quarters (79.4%, 95% CI 78.1‐80.7; 3947/4969) of survey respondents felt that restrictions could make someone feel bad or isolated. When asked if they would like more control over the content they viewed on social media, 87.8% (95% CI 86.7%‐88.8%; 3760/4281) of survey respondents answered yes.

#### Safety Features of Platforms

##### Censoring, Blurring, or Removal of Self-Harm Content

Almost all survey respondents had seen a post that had been censored, blurred, or marked with a warning (Table 3). More than 90% stated that they would be either likely or very likely to click on a censored post. Around a third of survey respondents answered that they would be less or much less likely to view a post with a self-harm specific content or trigger warning, while almost 40% said that they would be more likely or much more likely to view a post with a self-harm specific warning. Most survey respondents (89.1%, 95% CI 88.2%‐90%; 4449/4991) felt that having a post censored, blurred, or marked with a warning could draw more attention to the person who posted it.

We asked participants about their experiences of having their own posts censored or removed. Just 12.2% (95% CI 9.8%‐15.1%; 609/4992) of survey respondents had previously had a post censored or removed because of self-harm content; 21.1% (95% CI 18.7%‐23.8%; 1047/4955) of respondents had a post removed because of visible self-harm scars, and 21.1% (95% CI 18.7%‐23.7%; 1044/4952) of respondents had a post censored or removed that was related to recovery, raising awareness, or positive mental health. Having posts censored or removed for self-harm, visible scars, or positive mental health was experienced as either harmful or extremely harmful to participants at the time (Table 3). This was particularly the case for individuals who had posts censored or removed due to visible healed self-harm scars, where almost 90% of participants experienced this as either harmful or extremely harmful to them at the time. When asked, 84.6% (95% CI 83.5%‐85.7%; 4191/4951) of survey respondents stated that they would like the option to keep a post for themselves and simply have it removed from public view as opposed to being removed completely.

**Table 3.** Responses^a^ to survey questions related to censoring or blurring of images.

Question/response		Values, % (95% CI; n/N)
Viewing posts of others		
Have you ever seen a post that has been censored, blurred, or marked with a warning such as this? [Example image given]		
Yes		98.3 (97.9‐98.7; 4914/4997)
No		1.7 (0.2‐8.1; 83/4997)
Have you ever seen a censored post on an “explore” or “for you” page?		
Yes		73.0 (71.5‐74.4; 3644/4993)
No		27.0 (24.7‐29.5; 1349/4993)
How likely are you to click on a post that has been censored in this way?		
Very likely		64.3 (62.6‐65.9; 3212/4997)
Likely		27.2 (24.9‐29.7; 1361/4997)
Neither likely nor unlikely		5.4 (3.1‐9.0; 268/4997)
Unlikely		1.7 (0.2‐8.1; 83/4997)
Very unlikely		1.5 (0.1‐8.6; 73/4997)
If this post had a self-harm specific trigger warning/message, would this make you more or less likely to click on it?		
Much more likely		15.1 (12.6‐17.9; 753/4999)
More likely		21.4 (19.0‐24.0; 1069/4999)
Neither more nor less likely		28.5 (26.2‐31.0; 1427/4999)
Less likely		23.6 (21.2‐26.1; 1179/4999)
Much less likely		11.4 (9.0‐14.4; 570/4999)
Do you think this can draw more attention to the person who posted it?		
Yes		89.1 (88.2‐90.0; 4449/4992)
No		10.9 (8.4‐13.9; 542/4992)
Censoring of own posts		
Have you ever had any of your own posts censored or removed by a social media platform because of self-harm content?		
Yes		12.2 (9.8‐15.1; 609/4992)
No		87.8 (86.8‐88.7; 4383/4992)
If yes, how long after posting did this happen		
Less than 1 day		49.9 (44.1‐55.7; 299/608)
2‐14 days		34.5 (28.7‐42,0; 210/608)
15‐30 days		5.6 (0.9‐20.8; 34/608)
More than 30 days		5.3 (0.8‐21.0; 32/608)
More than 1 year		4.0 (0.2‐22.9; 24/608)
Not applicable		1.5 (0.5‐38.9; 9/608)
If yes, do you think having this post censored/removed was helpful or harmful to you at the time?		
Extremely harmful		22.6 (16.0‐30.9; 131/579)
Harmful		36.4 (30.0‐43.4; 211/579)
Neither helpful nor harmful		32.5 (25.9‐39.7; 188/579)
Helpful		6.0 (1.1‐21.0; 35/579)
Extremely helpful		2.4 (0.1‐30.0; 14/579)
Have you ever had a post censored or removed because of visible healed self-harm scars?		
Yes		21.1 (18.7‐23.8; 1047/4955)
No		78.9 (77.5‐80.1; 3908/4955)
If yes, do you think having this post censored/removed was helpful or harmful to you at the time?		
Extremely harmful		58.3 (54.3‐62.3; 599/1027)
Harmful		31.3 (26.3‐36.7; 321/1027)
Neither helpful nor harmful		9.7 (5.0‐17.7; 100/1027)
Helpful		0.4 (2.2‐60.7; 4/1027)
Extremely helpful		0.3 (3.0‐69.2; 3/1027)
Have you ever had a post censored or removed that was related to recovery, raising awareness, or positive mental health?		
Yes		21.1 (18.7‐23.7; 1044/4952)
No		78.9 (77.6‐80.2; 3908/4952)
If yes, do you think having this post censored/removed was helpful or harmful to you at the time?		
Extremely harmful		51.1 (46.8‐55.4; 528/1033)
Harmful		35.8 (31.0‐41.0; 370/1033)
Neither helpful nor harmful		10.6 (5.7‐18.3; 109/1033)
Helpful		1.6 (0.1‐25.2; 17/1033)
Extremely helpful		0.9 (0.7‐38.2; 9/1033)
Would you like the option to be able to keep a post for yourself and just have it removed from public view (as opposed to the post being removed completely)?		
Yes		84.6 (83.5‐85.7; 4191/4951)
No		15.4 (12.9‐18.2; 760/4951)

a Nonresponses not shown; percentages are displayed as a total of those who answered each question.

##### Reporting of Posts and Suspension of Accounts

More than half (58.6%, 95% CI 56.7%‐60.4%; 2663/4548) of survey respondents had reported a post created by someone else because of self-harm/suicide content. Of the individuals who had previously reported posts, over half (54.0%, 95% CI 51.5%-56.7%; 1437/2655) stated that this resulted in posts being removed “sometimes.”

Some platforms provide messaging for the individual if one of their posts is reported because someone is worried about them. One-fifth (19.7%, 95% CI 17.2%‐22.5%; 896/4540) of survey respondents had seen this sort of message before and 66.3% (95% CI 64.6%‐68.0%; 2981/4493) thought that this kind of message was helpful with 28.0% (95% CI 25.5%‐30.6%; 1256/4479) reporting that they would be more or much more likely to seek help after seeing this type of message. Half (50.6%, 95% CI 48.5%‐52.7%; 2277/4499]) of survey respondents felt that this sort of message could make someone feel isolated or lonely.

Just 11.0% (95% CI 8.5%‐14%; 525/4789) of survey respondents had ever had a social media account suspended for breaking the rules related to self-harm content. Of those respondents who had had their accounts suspended, only 34.2% (95% CI 27.4%‐41.7%; 179/523) received clear messaging from the platform about why the account had been suspended and 76.4% (95% CI 71.9%‐80.4%; 399/522) stated that having their account suspended was either harmful or extremely harmful to them at the time.

##### Help Messaging and Signposting

More than three-quarters (77.9%, 95% CI 76.5%‐79.2%; 3619/4646) of survey respondents had seen messaging signposting to sources of help. When asked, 20.8% (95% CI 18.3%‐23.5%; 962/3665) said that this sort of message would stop them searching for self-harm content and 34.0% (95% CI 31.7%‐36.4%; 1577/4638) had contacted one of the professional sources of help. More than half (52.8%, 95% CI 50.8%‐54.8%; 2179/4614) of survey respondents felt that these sources of help are not relevant to them. One-fifth (19.4%, 95% CI 16.9%‐22.2%; 899/4645) of survey respondents said that they would be likely or very likely to try one of the tips for support.

More than three-quarters (79.8%, 95% CI 78.4%‐81.2%; 3327/4165) of survey respondents would be more likely to seek/accept help if they could access a messaging or texting service instead of a helpline, 56.7% (95% CI 54.7%‐58.7%; 2362/4165) would be more likely to seek/accept help specific to their local area, and 46.0% (95% CI 43.8%‐48.3%; 1917/4165) would prefer having the option to have someone contact them.

### Possible Changes to Platform Features

**Figure 1.**
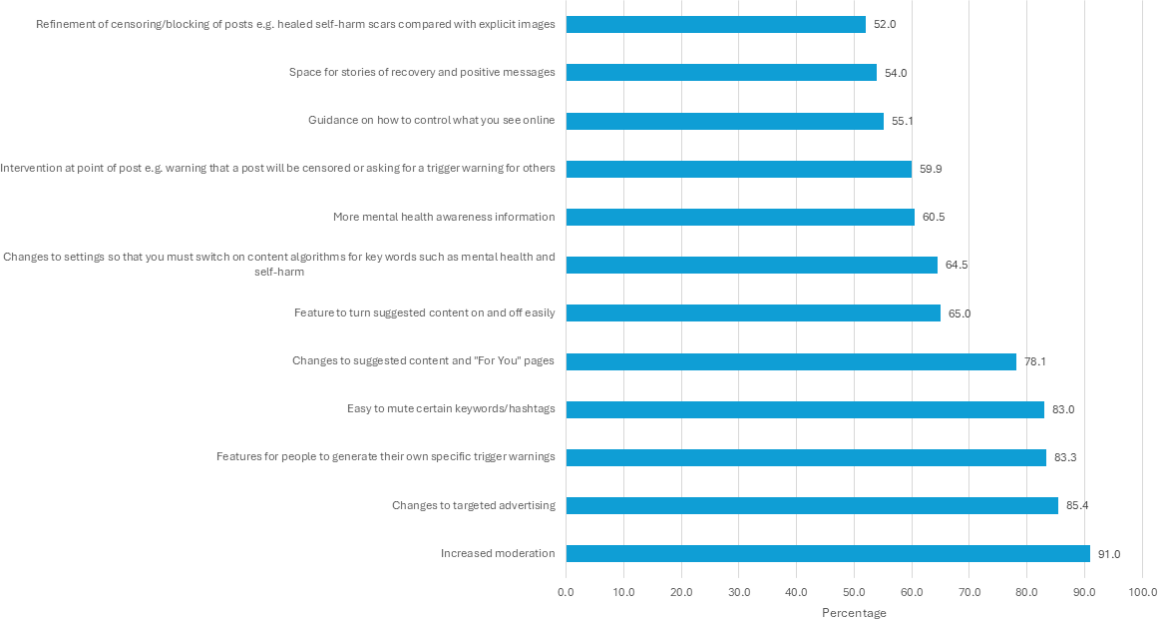
Responses to the question “Which of these features would have a positive impact on content? Tick all that apply.” Full statistical data are presented in Multimedia Appendix 2.

The patient and participant involvement (PPI) focus groups generated a number of suggestions for how to improve current features of platforms, and we put these to our survey respondents (Figure 1). Every suggestion included in the survey was said to have the potential for a positive impact by more than half of the respondents. The most popular options were increased moderation, changes to targeted advertising, features for people to generate their own specific content warnings, and easy-to-mute keywords/hashtags.

### In-Depth Interviews

#### Overview

A total of 17 participants aged 16‐56 years (average 23.8 years) took part in the in-depth interviews. Data saturation (no new themes data/concepts emerged) was reached during this process. More than half were aged 18‐24 years (Table 2) and 16 out of 17 had a history of self-harm.

Thematic analysis of the interviews revealed 13 subthemes organized into 7 overarching thematic areas (Table 4; full details in Multimedia Appendix 4; integration with quantitative data in Multimedia Appendix 5; thematic map in Multimedia Appendix 6).

**Table 4.** Themes, subthemes, and examples identified from analysis of in-depth interviews.

Theme and subtheme	Example
1.0 Experiences of viewing self-harm/suicide-related content	
Nature of content including whether pictures/text, platform, and whether this was searched or suggested content	“I initially found it under the ‘depressed’ as I wouldn’t have really known what self-harm was. It then became something I was exposed to irregularly because I was considering doing it.” [Interview Participant 15]
Age and safety of younger people	“I think I first started seeing self-harm on Instagram when I first got it, so when I was 12. Explicit photos of people’s self-harm injuries mostly.” [Interview Participant 2]
Effect of self-harm content on mood or behavior	“Accessing written descriptions of self-harm also gave me more ‘ideas’ of ‘different’ ways to self-harm that I personally wouldn’t have thought of if I never came across that content in the first place.” [Interview Participant 1]
2.0 Online communities	
Importance of community support	“It’s strange because even though there is harmful content on it, I find a lot of enjoyment and entertainment out of it! I think it can be a helpful/community space is used correctly, I’ve made a lot of friends over social media ☺️” [Interview Participant 7]
Accounts separate to their own personal account dedicated to mental health or self-harm content, often referred to as a recovery account	Researcher: “Did you find this [having a recovery account] helpful?” Participant 5: “That’s a difficult question because the answer is yes and no. Helpful to feel less alone but kept me caught up with mental illness as my identity and getting worse as it sparked a competitive edge in me.”
3.0 Experiences of self-harm/suicide content restrictions	
Restrictions effective/necessary	“At that time, none of those sorts of accounts/pages were private or restricted, whereas now I’m almost certain it would be harder to access, or warnings would be given beforehand etc.” [Interview Participant 6]
Restrictions ineffective	“I think it become worse to be honest. It’s easier to find although it does come with a warning now if you search for self-harm.” [Interview Participant 3]
Restrictions making someone feel bad or isolated	“Stopping all discussion may be counter intuitive and remove lifelines for some people.” [Interview Participant 14]
4.0 Censoring, blurring, or removing content	
Role of content/trigger warnings and whether this is an effective barrier against harmful content	“I think on Twitter a lot of individuals who discuss content will state, ‘TW/’ and then whatever it may be, so it allows the individual to make a choice. It could definitely be beneficial because not everything will provoke the same responses in people, and I have found that sometimes if I proceed to see a post I’m like ‘why did they sensor this’ etc.” [Interview Participant 6]
Experiences of having own posts blurred, censored, or removed for self-harm content, visible healed scars, mental health, and recovery	“I follow a young girl who shares her Tourette’s journey and often has her videos removed because of her tics/if she is swearing etc, it can cause problems for her because she is advocating awareness and education for Tourette’s, which there is a lot of misconception of, but it’s misunderstood by guidelines.” [Interview Participant 6]
5.0 Reporting of posts	
Procedure when a post is reported	“...and for social media companies to actually investigate those reports, because it seems that even when you report that, the post or account stays up.” [Interview Participant 4]
Reaching out to someone as alternative to reporting posts	“I very regularly reach out, it’s very regular that actual self-harm is done by an ‘anonymous’ user, so you don’t know whose actually behind the profile so it’s difficult to contact anyone.” [Interview Participant 15]
6.0 Help messaging and signposting	
Help messaging and help seeking	“I am actually quite a likely person because I want help that is accessible to me-that isn’t just cahms or going through the NHS-I know of SHOUT which I have used before which is really helpful.” [Interview Participant 7]
Nature of messaging	“I’ve seen ones on Instagram-I’ve seen ads on tiktok saying resources where you can gain access to therapists online/phone numbers but those are usually American so it doesn’t really matter to people in the UK.” [Interview Participant 7]
7.0 Possible changes to platform features	
Age restrictions and verification	“...things like proving your age like YouTube has done-I couldn’t watch a Rihanna music video until I turned 18 due to the age restrictions haha-you had to verify with a bank account or something I think. So maybe better verification like that if harmful content is gonna possibly be available.” [Interview Participant 7]
More control over suggested content	“I can’t seem to find a feature to make it not appear on my for you page.” [Interview Participant 11]
More positive and uplifting content/content to raise awareness	“...I think that instead of suggesting accounts similar to what the person is seeking out, they could instead recommend accounts focusing on recovery.” [Interview Participant 4]
Guidance for posters/restrictions when creating a post	“It would be useful if post had to be tagged with any major warnings, maybe out of a list, when posted. So instead of just being able to block posts explicitly tagging self-harm, you could block any post where it’s flagged as a warning.” [Interview Participant 2]
Improvements to signposting	“...instant support would be better like a message support.” [Interview Participant 5]

#### Theme 1.0: Experiences of Viewing Self-Harm/Suicide Content

#### Theme 1.1: Nature of content

Interview participants described viewing memes, text, photos, and videos with content ranging from community/support to graphic self-harm images and videos, instructions on methods and materials that were described as triggering.

##### Theme 1.2: Age and Safety of Younger People

Participants emphasized the need for better regulation for young people, with current age restrictions said to be easy to bypass.

*I was 11 - understandably under the age of Instagram joining age however there wasn’t a lot to protect children from joining.*

[Interview Participant 15]

##### Theme 1.3: Effects of Self-Harm Content on Mood or Behavior

Viewing self-harm content was said to have a range of effects on mood and behavior, with some participants describing worsening self-harm and lowering of mood, while others talked about self-harm content having a varying impact depending on their current mood and well-being.

*Depending on my mood and the content, it can have a range of effects. If I am not in a good mindset and not prepared for such content it can trigger a flare up in my symptoms, sometimes very severe, and can cause distress. Usually these days, I can tolerate and even enjoy such content. I also find some posts very helpful and meaningful as I follow many self-help and psychiatry pages.*

[Interview Participant 10]

### Theme 2.0: Online Communities

#### Theme 2.1: Importance of Community Support

The importance of online communities was discussed at length. There were discussions about gaps in service provision and an absence of support in schools and health care settings and turning to online communities for support.

*It’s especially more difficult when topics like self-harm are almost taken lightly in schools/are stigmatised [...] I think it’s not to do with the discussion it’s the authority, for example - I came to my school and told them my best friend is self-harming I’m really worried for her well-being and they didn’t do anything/take it seriously - They tell us that don’t do this get help but when you try to get that help it doesn’t really work.*

[Interview Participant 7]

#### Theme 2.2: Dedicated Recovery Accounts

Participants talked about positive and negative aspects of both maintaining and following dedicated mental health/self-harm or recovery accounts. Following these accounts was described as both an inspiration for recovery and fueling comparison over whether the individual’s own recovery was flawed in comparison. Negative experiences of following accounts dedicated to mental health/self-harm, where there was little recovery-oriented content, were described. Maintaining these accounts was described both in terms of reducing isolation and contributing to mental illness forming part of their identity. Discussions highlight the need for better guidance for content creators.

### Theme 3.0: Experiences of Self-Harm/Suicide Content Restrictions

We asked participants about the impact of the change in policies in 2019. Responses can be broadly divided into restrictions being effective or necessary, restrictions being ineffective, and the potential for restrictions to make someone feel bad or isolated (described below).

#### Theme 3.1: Restrictions Effective/Necessary

Participants talked about self-harm content before the current restrictions were put in place. Many expressed that the change was needed and that there have been both decreases in the amount of negative content alongside increases in positive and uplifting content.

*In general, when I was younger access to content displaying self-harm was more prominent but with the trigger/content warnings on things now it’s a little more regulated. The sort of content being-what pills to take that will kill you, videos of people cutting themselves (this was mainly on YouTube early 2010‐15) from what I remember and I’m sure there was more out there Twitter still is particularly bad for pro-anorexia/food self-harm.*

[Interview Participant 7]

*It has 100% improved and is more pushed now - If I’m in a depressed mindset I try and look for uplifting posts. If you want to find self-harm content, you really have to look for it.*

[Interview Participant 15]

#### Theme 3.2: Restrictions Ineffective

Some participants described little change in content following restrictions. Others discussed the ease of bypassing current restrictions and that self-harm content is still easy to find.

*I also think imposing extreme limits can be equally damaging, as people find ways to get around it rather than being able to share in a more informed way. For example, people will use “sewerslide” to refer to suicide, meaning the content does not get reviewed but is then also freely accessible to anyone.*

[Interview Participant 6]

#### Theme 3.3: Restrictions Making Someone Feel Bad or Isolated

Removal of content and restrictions were said to risk taking away a space to talk about experiences and increasing isolation for individuals who depend on these communities.

*On the flip side of that I think removing any mention of self-harm would be damaging, as young people experiencing it could feel isolated and then less willing to speak to someone, so I think there are definitely some positives to social media in being able to share appropriate and helpful information around sensitive topics.*

[Interview Participant 6]

### Theme 4.0: Censoring or Blurring of Self-Harm Images

A distinction was made between the need to censor images of fresh self-harm/graphic imagery and the risk of increasing stigma by censoring healed self-harm scars. Participants discussed whether censoring is an effective barrier to content, which may increase distress as well as experiences of having their own content censored or removed.

#### Theme 4.1: Role of Content/Trigger Warnings and Whether This Is an Effective Barrier Against Harmful Content

Blurring or warning messages on posts were described as enabling individuals to make a choice about whether to view content.

*I scroll past I try not to set myself back by triggering content.*

[Interview Participant 17]

It was emphasized that for content warnings to be effective they needed to be specified to self-harm/suicide content.

#### Theme 4.2: Experiences of Having Own Posts Blurred, Censored, or Removed

No interview participants had experienced having their own content removed, but some spoke of the experiences of others where content related to recovery or raising awareness was repeatedly removed for some accounts.

### Theme 5.0: Reporting Posts

#### Theme 5.1: Procedures When a Post Is Reported

Participants described reporting posts for self-harm content and that posts were sometimes removed but not always. Frustration was expressed at the lack of human review, having to report posts multiple times, and automated tools not adequately detecting harmful content.

*...they say there is nothing wrong with it. It’s sad to see their algorithms not working it would be good if a human verified it.*

[Interview Participant 17]

#### Theme 5.2: Reaching Out to Someone as Alternative to Reporting Posts

Participants described often reaching out to others if they were worried about them as an alternative to reporting posts to platforms. Many expressed that it was more meaningful to hear from a friend than platform messaging. Guidance on how to reach out to someone would better equip them for these conversations.

*...I think a friend reaching out personally would be more helpful in most cases. The help messages only seems applicable if it’s somebody you're not close to flagging concern...I know that when I've been worried about friends, I haven't always known what to say.*

[Interview Participant 2]

*...a way to reach them would be better this would help on individuals we know or someone we feel we can help out*.

[Interview Participant 8]

### Theme 6.0: Help Messaging and Signposting

We gave participants examples of help messaging from platforms in response to self-harm/suicide-related content or searches, including signposting to sources of help as well as tips for support such as reaching out to a friend or taking a break from the screen. Some responded positively to the range of help options available, while others talked about the international nature of helplines and an absence of relevance to the United Kingdom. Participants talked about the potential for messages and tips for support to be experienced positively, depending on the level of individual distress.

*I think it’s a really fine line between being condescending and providing helpful suggestions, I can imagine that for some people with moderate depression being prompted to take some time away from the screen could be helpful, I think if you have reached the point of planning to kill yourself being encouraged to “explore activities that make you happy” is unlikely to be all that helpful*.

[Interview Participant 13]

### Theme 7.0: Possible Changes to Platform Features

Participants discussed the following possible changes that would improve platform safety.

#### Theme 7.1: Age Restrictions and Verification

Suggestions for improved safety features for young people included stricter age verification procedures, parental controls, and separate features for younger individuals such as blocking sensitive content altogether rather than blurring or marking with a warning.

#### Theme 7.2: More Control Over Suggested Content

Participants discussed the current features and how these were not intuitive or user-friendly.

*It feels invasive that I am bombarded with what Facebook thinks I want. I feel hounded by it.*

[Interview Participant 14]

*I’ve had a look but can’t seem to find anything to stop me from seeing that.*

[Interview Participant 17]

Participants highlighted the need for easy-to-use filters to be able to filter out content that they did not want to see.

*I think it would be helpful if Instagram allowed you to select filters where you can filter out the types of contact you don’t want to see on your explore page...I think you can sort of suggest a post that you don’t like and want to see “less” of. But I think it would be more beneficial if you could highlight specific categories like “self-harm” “eating disorders” “diets” “depression” etc.*

[Interview Participant 1]

Suggestions were also made that certain types of content should be hidden by default and that users should have to manually turn on algorithms to see this content.

#### Theme 7.3: More Positive and Uplifting Content/Content to Raise Awareness

Changes to suggested content with algorithms pushing more positive and uplifting content and raising awareness were also discussed.

*Potentially the best method could be to go from the other way, and integrate promotions for support into mainstream feeds so people know where they can go or who they can talk to, for example. I think that if someone was determined enough they could probably find what they are looking for, so advocating the support they could access may be bunker successful/reduce a lot of stigma*.

[Interview Participant 6]

#### Theme 7.4: Guidance for Posters/Restrictions When Creating a Post

There were conversations about posting about self-harm to find a connection with others and that many who post do not intend harm to others.

*I think, most of the time, people are just misguided on the best approach. Most people have good intentions, and mean no harm.*

[Interview Participant 10]

The need for guidance on how to talk about self-harm in a safe way, including information on what to post, the nature of images, and language to use, as well as additional prompts and options from the platform at the point of post, was discussed. Lists of keywords that would be easy to block or filter were also suggested, although it was highlighted that these terms should only be used to block content and should not be searchable.

#### Theme 7.5: Improvements to Signposting

Participants discussed a preference for messaging services over helplines and discomfort with having to talk to someone on the phone. The option for a live chat available on the platform in the moment of distress was also suggested, as was an automated message sent to the messaging part of the app.

Participant: *Maybe live chat people who could reach out if there was a concerning post and give them someone else to speak to. I think if people search for triggering content the only stuff that should come up is helplines.*

Researcher: *Is this something you would have engaged with?*

Participant 15: *Yes 100%.*

## Discussion

### Principal Findings

This study set out to gain both quantitative and qualitative data to better understand the perspectives of social media users on safety features related to self-harm and suicide content. To our knowledge, this is the first study to gain perspectives of individuals with a history of self-harm related to social media safety features. Through a survey and in-depth interviews, we gained insights into individuals’ experiences of self-harm and suicide content on social media. Half of survey respondents felt that the introduction of additional features by platforms in 2019 made social media safer, with positive experiences of some features including increases in uplifting content. Content that was potentially harmful was said to not always be effectively censored or promptly removed. This existed alongside distress caused by the censoring of images where self-harm scars are visible and where accounts were suspended. The messaging when content was removed or censored was not felt to be sufficiently clear. Changes to current features to improve safety from the perspectives of individuals included increasing safety and protection for younger people; refinements to policies related to censoring of content with additional care needed by platforms to allow safe discussion of self-harm and sharing of stories of recovery; more control over content with intuitive, easy-to-use controls; and improvements to signposting information with tailoring by location and availability of live chat or messaging options.

Previous research using content analysis of Tweets before and after the change in platform policies found that some harmful content was still present and that positive mental health or “self-harm clean” stories were also being censored [8]. The current study extends this work going beyond an analysis of content to gain insights from the perspectives of individuals. Participants report that graphic content is now harder to find, although it is still present. The potentially harmful effects of censoring or removing posts related to recovery, positive mental health, or where self-harm scars are visible were highlighted. This is in keeping with previous evidence of the beneficial effects of media narratives of hope and recovery from suicidal crises [30]. Offline gaps in services such as mental health services, as well as a lack of societal understanding of self-harm, can lead young people to seek support from one another while waiting for specialist care [24,31]. Having open conversations related to mental health on social media can benefit users [32], and in-depth interview participants here describe the value of online communities. This supports recent calls to ensure that any restrictions put in place by social media platforms are evidence based [7] with an awareness of the risks of imposing restrictions in online spaces and how this could impede vital sources of support and increase stigma [24,33].

### Strengths and Limitations

Online recruitment allowed for a rapid, low-cost engagement and lowered some barriers to participation. However, this was a convenience sample and as such is limited in terms of generalizability. Participants here were largely female and younger than 25 years, and most had a history of self-harm. We intended to gain insights from those both with and without a history of self-harm; however, in both the survey and in-depth interviews, the majority of individuals had a history of self-harm, meaning we could not capture differences between groups. Small numbers of individuals who had their posts censored or removed and who subsequently answered items on the impact of this have resulted in wide CIs for some items, and results should be interpreted accordingly.

Whether people are at greater risk of self-harm because they engage with such content online, or rather they end up engaging with it because they were at greater risk of self-harm to begin with cannot be determined based on this study. While we have attempted to offer a nuanced and up-to-date analysis, the social media dynamic is constantly changing and evolving [11]. As such, there is a need for ongoing research as papers become outdated quickly.

### Implications

This work was undertaken as part of the Samaritans online excellence program, and findings have fed into evidence for the Online Harms Bill [34]. The survey developed by the lived experience group has informed further studies related to social media safety (eg, [35]).

This research supports evidence of the potential risks of imposing restrictions on online spaces [33]. While there have been calls for tighter legislation for platforms, there is a lack of evidence-based recommendations [11]. There are concerns over the evidence base underpinning social media bans for those younger than 16 years, with the potential for unintended harms and a missed opportunity for a more nuanced approach to improving safety [36]. Alternative approaches may include harm minimization and co-designed education initiatives to equip young people with the skills and knowledge to safely navigate the online world [37].

Since the date of these interviews, platforms have continued to develop safety features. For example, TikTok now asks for age verification with an identification card in countries where identification cards are mandatory (this is not the case in the United Kingdom) and offers limited features for younger users. Algorithms have also been adjusted so that posts related to topics such as mental health are interspersed with other content [38]. The current work suggests that location-specific signposting to both messaging and other services and proactive outreach may have the potential to improve help-seeking in young people with experience of self-harm. Participants also described reaching out to others when seeing a distressed post on social media and discussed the potential utility of educating individuals (both those viewing content and content creators) on how to talk about self-harm in a safe way. Participants suggested that platforms provide tools and prompts to help individuals make informed decisions about what they post. Such initiatives could build on existing resources (eg, [39]).

### Conclusions

This study offers novel insights into how social media users with a history of self-harm experience social media safety features related to self-harm and suicide content. Findings highlight that while restrictions may reduce harmful content, they may also unintentionally limit recovery narratives, images where self-harm scars are visible, and community support. This work underscores the importance of user involvement in developing safety features and the need for ongoing evaluation to ensure supportive online environments while minimizing harm.

## Supplementary material

10.2196/73343 Multimedia Appendix 1 Focus group design and analysis.

10.2196/73343 Multimedia Appendix 2 Summary of all survey responses.

10.2196/73343 Multimedia Appendix 3 Summary of item nonresponse.

10.2196/73343 Multimedia Appendix 4 Themes, subthemes, and further examples identified from analysis of in-depth interviews.

10.2196/73343 Multimedia Appendix 5 Integration of survey and interview data.

10.2196/73343 Multimedia Appendix 6 Thematic map.

## Abbreviations

ADP: Adolescent Mental Health Data Platform
CHERRIES: Checklist for Reporting Results of Internet E-Surveys
JARS-Mixed: APA Journal Article Reporting Standards for Mixed Methods Research
NCSR: National Centre for Suicide Prevention and Self-Harm Research
PPI: patient and participant involvement
SHARE UK: Self-Harm Research UK
