# Supplementary material for: Experiences of Social Media Platforms’ Policies and Restrictions Related to Self-Harm Content: Mixed Methods Study
Source: J Med Internet Res. 2026 Aug 6;28:e73343. doi: 10.2196/73343 (PMC13446788; doi:10.2196/73343)
Supplement: Supplementary file 1 [file jmir-v28-e73343-s006.pdf]

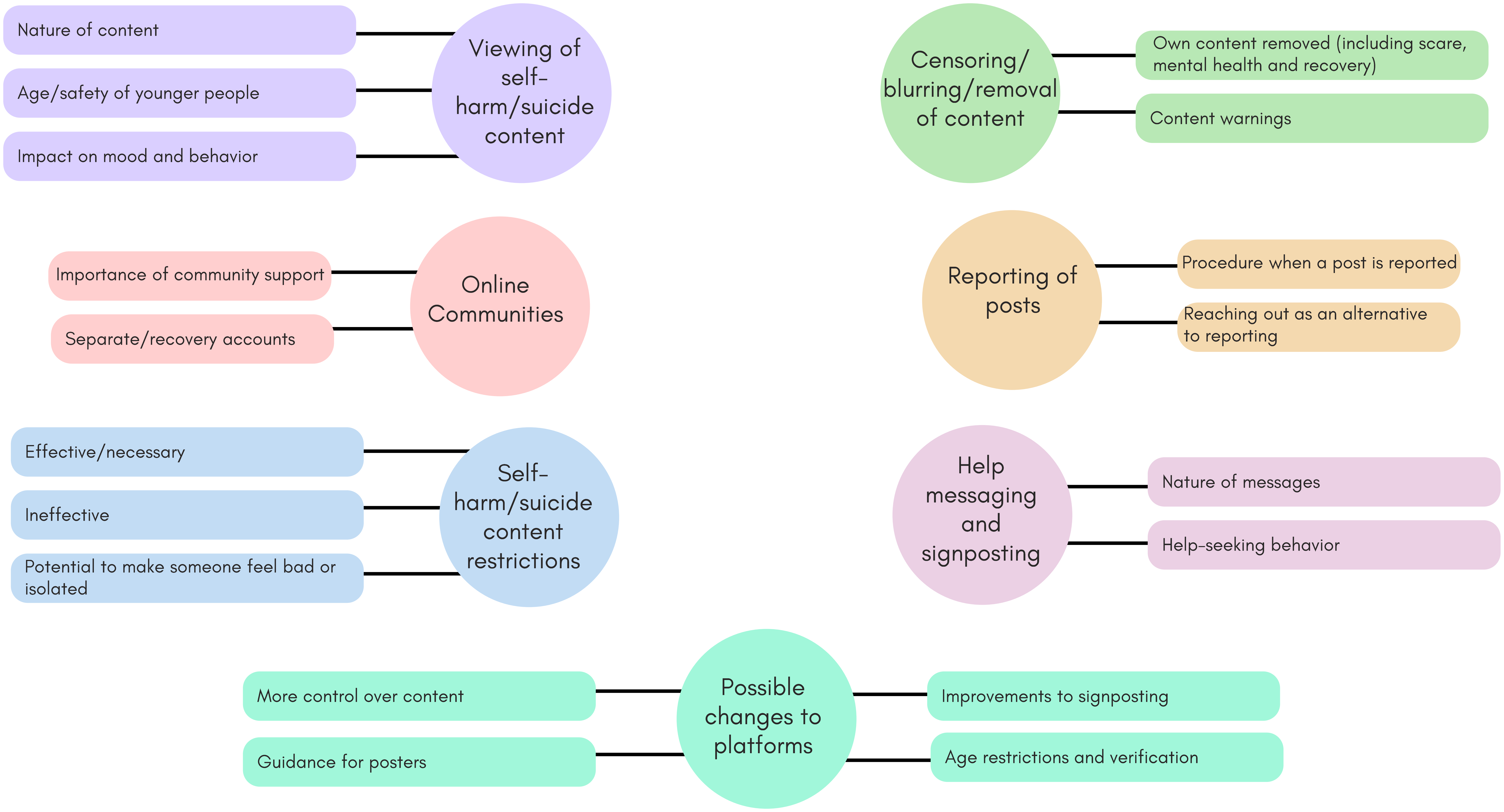
Viewing of self-harm/suicide content
Nature of content
Age/safety of younger people
Impact on mood and behavior
Censoring/blurring/removal of content
Own content removed (including scare, mental health and recovery)
Content warnings
Online Communities
Importance of community support
Separate/recovery accounts
Reporting of posts
Procedure when a post is reported
Reaching out as an alternative to reporting
Self-harm/suicide content restrictions
Effective/necessary
Ineffective
Potential to make someone feel bad or isolated
Help messaging and signposting
Nature of messages
Help-seeking behavior
Possible changes to platforms
More control over content
Guidance for posters
Improvements to signposting
Age restrictions and verification
