# Supplementary material for: Experiences of Social Media Platforms’ Policies and Restrictions Related to Self-Harm Content: Mixed Methods Study
Source: J Med Internet Res. 2026 Aug 6;28:e73343. doi: 10.2196/73343 (PMC13446788; doi:10.2196/73343)
Supplement: Supplementary file 2 [file jmir-v28-e73343-s001.docx]

# Focus group design and analysis.

A series of focus groups were run to inform the national survey and in-depth interviews.

## Participants

Participants aged 16 and over, with and without a history of self-harm were recruited. Self-harm refers to any intentional act of self-injury or poisoning independent of motivation or suicidal intent [1]. This definition is necessarily very general as the motivation or degree of suicidal intent is difficult to assess and may vary between individuals and over time. A history of self-harm was based on self-report from participants at the onset of the study.

## Data Collection

Three online focus groups were conducted between 17.01.2022-20.01.2022. The purpose of these focus groups was to inform the topics covered in the survey and in-depth interviews. The focus groups contained a maximum of six people, lasted around one hour and took place via Zoom. Holding focus groups online lowers many barriers to taking part, removing the need for travel and allowing for a greater level of anonymity as participants could choose not to turn their cameras on if preferred.

Focus groups were conducted by both a senior researcher (AM) and a clinician (AJ).

### Data analysis

Online focus groups were digitally recorded with consent and transcribed verbatim from recordings. Each individual was given a participant number to replace their name and any names or identifiable information present in transcripts were masked. Completed transcripts were reviewed to ensure participant anonymity was retained. Data from focus groups was analysed utilising cross-case thematic analysis [2].

A coding frame was developed based on previous literature [3-10, 11{Marchant, 2020 #335, 12] and indictive generation of codes. Data were sorted thematically, using a code-and-retrieve system [13]. All focus-group transcripts were reviewed by two members of the study team (AM, MS). A coding frame was developed through a process of reading and re-reading interviews, inductive generation of codes and repeated discussions (AM, AJ, MS). Final coding was conducted by one member of the study team (AM) and cross-checked by a senior member of the team (AJ).

Themes were organised using Nvivo 12. The number of individuals referring to each theme was calculated.

### Development of survey and in-depth interview schedules

Ten participants aged 18 to 44 years, (5 female, 4 male, 1 other) took part in online focus groups to inform the topics to be studied and questions asked in the survey and interviews. Focus groups followed a semi-structured format with topics including: what do participants feel research should cover; are there any platforms that require special consideration; experiences of messaging of platforms and areas of concern; the role of trigger warnings; any special considerations needed for images; other features of platforms and, key principles that platforms should follow.

Discussions centred notably around images and videos, reflecting the way social media has evolved and moved away from forums and written content. Five main topic areas for further exploration were identified (Table 1). These topics formed the basis of the survey and in-depth interview schedule. The importance of balance and the positive and negatives of both social media and the restrictions around self-harm and suicide content was also repeatedly discussed. Participants talked about the use of social media as a space for journalling and community support as well as highlighting times when restrictions have been harmful. This was an important consideration in the development of the survey and interviews and care was taken to gain a balanced view.

## References

1.	NICE, *Self-harm: assessment, management and preventing recurrence (NICE guideline NG225)*. 2022, National Collaborating Centre for Mental health

2.	Deterding, N.M. and M.C. Waters, *Flexible coding of in-depth interviews: A twenty-first-century approach.* Sociological methods & research, 2021. **50**(2): p. 708-739.

3.	Lavis, A. and R. Winter, *# Online harms or benefits? An ethnographic analysis of the positives and negatives of peer‐support around self‐harm on social media.* Journal of child psychology and psychiatry, 2020. **61**(8): p. 842-854.

4.	Seko, Y., et al., *On the creative edge: Exploring motivations for creating non-suicidal self-injury content online.* Qualitative health research, 2015. **25**(10): p. 1334-1346.

5.	Jacob, N., R. Evans, and J. Scourfield, *The influence of online images on self-harm: A qualitative study of young people aged 16-24.* Journal of Adolescence, 2017. **60**: p. 140-147.

6.	Record, R.A., K. Straub, and N. Stump, *# Selfharm on# Instagram: examining user awareness and use of Instagram’s self-harm reporting tool.* Health communication, 2019.

7.	Carlyle, K.E., et al., *Suicide conversations on Instagram™: contagion or caring?* Journal of Communication in Healthcare, 2018. **11**(1): p. 12-18.

8.	Miguel, E.M., et al., *Examining the scope and patterns of deliberate self-injurious cutting content in popular social media.* Depression and Anxiety, 2017. **34**(9): p. 786-793.

9.	Arendt, F., S. Scherr, and D. Romer, *Effects of exposure to self-harm on social media: Evidence from a two-wave panel study among young adults.* New Media & Society, 2019. **21**(11-12): p. 2422-2442.

10.	Moreno, M.A., et al., *Secret Society 123: Understanding the Language of Self-Harm on Instagram.* Journal of Adolescent Health, 2016. **58**(1): p. 78-84.

11.	Shanahan, N., C. Brennan, and A. House, *Self-harm and social media: thematic analysis of images posted on three social media sites.* BMJ open, 2019. **9**(2): p. e027006.

12.	Marchant, A., et al., *A systematic review of the relationship between internet use, self-harm and suicidal behaviour in young people: The good, the bad and the unknown.* PLoS one, 2017. **12**(8): p. e0181722.

13.	Coffey, A. and P. Atkinson, *Concepts and coding.* Making sense of qualitative data: Complementary research strategies, 1996: p. 26-53.
