# Supplementary material for: Experiences of Social Media Platforms’ Policies and Restrictions Related to Self-Harm Content: Mixed Methods Study
Source: J Med Internet Res. 2026 Aug 6;28:e73343. doi: 10.2196/73343 (PMC13446788; doi:10.2196/73343)
Supplement: Supplementary file 3 [file jmir-v28-e73343-s002.docx]

# Summary of all survey responses

| Question/Response | %(95% CI) | |  |
| --- | --- | --- | --- |
| How old were you when you first saw self-harm or suicide related content on social media? | | | |
| 10 years or younger | 17.4(15-20.1);921 | |  |
| 11-14 years | 68.8(67.3-70.3);3639 | | |
| 15-16 years | 9.3(7-12.3);493 | |  |
| 17-18 years | 1.4(0.1-8.5);72 | |  |
| 19-24 years | 1.4(0.1-8.4);73 | |  |
| 25 years or older | 1.7(0.2-7.8);91 | |  |
|  |  |  |  |
|  |  |  |  |
| If yes [to viewing self-harm content] on which platform (tick all that apply) | | |  |
| Instagram | 79.8(78.5-81; 4219) | |  |
| Facebook | 16.3(13.9-19; 862) | |  |
| Tumblr | 36.2(34-38.4; 1912) | |  |
| TikTok | 70.2(68.7-71.6; 3711) | | |
| Twitter | 39.4(37.3-41.6; 2086) | | |
| Pinterest | 18.5(16.2-21.1; 979) | |  |
| MySpace | 2.6(0.7-7.3; 135) | |  |
| GoogleImages | 34.1(32-36.4; 1805) | |  |
| Other | 15.1(12.7-17.8; 799) | |  |
|  |  |  |  |
| Have you ever had more than one social media account and kept one or more just for mental health/self-harm content? | | | |
| Yes | 39.2(37.1-41.3; 2065) | | |
| No | 60.8(59.1-62.5; 3208) | | |
|  |  |  |  |
| Have you ever viewed self-harm or suicide content on social media that you did not search for, for example on the 'explore' or 'For you' pages? | | | |
| Yes | 82.9(81.7-84; 4376) | |  |
| No | 17.1(14.8-19.8; 904) | |  |
|  |  |  |  |
| If Yes on which platform? Tick all that apply |  |  |  |
| Instagram | 62.6(60.8-64.4; 2737) | | |
| Facebook | 9.1(6.5-12.5; 397) | |  |
| Tumblr | 19.8(17.2-22.7; 866) | |  |
| TikTok | 74.7(73.2-76.2; 3266) | | |
| Twitter | 19.6(17-22.4; 856) | |  |
| Pinterest | 8.8(6.3-12.2; 385) | |  |
| MySpace | 1.1(0-11.3; 46) | |  |
| GoogleImages | 6.3(3.9-10.1; 277) | |  |
| Other | 5.4(3-9.3; 235) | |  |
|  |  |  |  |
|  |  |  |  |
| When I see online content about self-harm this is: |  |  |  |
| Always/mostly accidental (e.g. shared with me by someone else) | 18.4(16-21; 972) | |  |
| Always/mostly intentional | 8.2(5.9-11.3; 433) | |  |
| I'm not sure/neither accidental nor intentional | 28.6(26.3-31; 1511) | |  |
| Often accidental | 24.9(22.6-27.4; 1316) | | |
| Often intentional | 19.9(17.5-22.5; 1051) | | |
|  |  |  |  |
| Usually, seeing, sharing or posting online content about self-harm: | |  |  |
| Improves my mood | 1.5(0.1-8.1; 80) | |  |
| Does not change my mood | 10.8(8.4-13.7; 570) | |  |
| Worsens my mood | 34.9(32.7-37.1; 1844) | | |
| Depends on my emotions at the time | 52.8(50.9-54.6; 2788) | | |
|  |  |  |  |
|  |  |  |  |
| As a consequence of seeing self-harm content online, have you ever performed the same (or very similar) self-harm? | | | |
|  |  |  |  |
| Never | 23.2(20.8-25.7; 1163) | | |
| Sometimes | 60(58.2-61.7; 3012) | |  |
| Often | 16.8(14.4-19.6; 846) | |  |
|  |  |  |  |
| As a consequence of seeing self-harm content online, have you ever self-harmed more or more severely? | | | |
|  |  |  |  |
| Never | 24.2(21.8-26.7; 1216) | | |
| Sometimes | 51.6(49.6-53.5; 2590) | | |
| Often | 24.2(21.8-26.7; 1216) | | |
|  |  |  |  |
| Have you ever seen a post that has been censored, blurred or marked with a warning such as this? | | | |
| Yes | 98.3(97.9-98.7; 4914) | | |
| No | 1.7(0.2-8.1; 83) | |  |
|  |  |  |  |
| Do you think this can draw more attention to the person who posted it? | | |  |
| Yes | 89.1(88.2-90; 4449) | |  |
| No | 10.9(8.4-13.9; 542) | |  |
|  |  |  |  |
| How likely are you to click on a post that has been censored in this way? | | |  |
| Very likely | 64.3(62.6-65.9; 3212) | | |
| Likely | 27.2(24.9-29.7; 1361) | | |
| Neither likely or unlikely | 5.4(3.1-9; 268) | |  |
| Unlikely | 1.7(0.2-8.1; 83) | |  |
| Very unlikely | 1.5(0.1-8.6; 73) | |  |
|  |  |  |  |
| If this post had a self-harm specific trigger warning/message would this make you more or less likely to click on it? | | | |
| Much more likely | 15.1(12.6-17.9; 753) | |  |
| More likely | 21.4(19-24; 1069) | |  |
| Neither more or less likely | 28.6(26.2-31; 1427) | |  |
| Less likely | 23.6(21.2-26.1; 1179) | | |
| Much less likely | 11.4(9-14.4; 570) | |  |
|  |  |  |  |
| Have you ever seen a censored post on an 'explore' or 'for you page'? | | |  |
| Yes | 73(71.5-74.4; 3644) | |  |
| No | 27(24.7-29.5; 1349) | |  |
|  |  |  |  |
| Have you ever had any of your own posts censored or removed by a social media platform because of self-harm content? | | | |
| Yes | 12.2(9.8-15.1; 609) | |  |
| No | 87.8(86.8-88.7; 4383) | | |
|  |  |  |  |
| If yes, how long after posting did this happen |  |  |  |
| 15-30 days | 5.7(0.9-20.8; 34) | |  |
| 2-14 days | 35.1(28.7-42; 210) | |  |
| Less than 1 day | 49.9(44.1-55.7; 299) | |  |
| more than 1 year | 4(0.2-22.9; 24) | |  |
| more than 30 days | 5.3(0.8-21; 32) | |  |
| Not applicable | 1.5(0.5-38.9; 9) | |  |
|  |  |  |  |
| If yes, do you think having this post censored/removed was helpful or harmful to you at the time? | | | |
| Extremely harmful | 22.6(16-30.9; 131) | |  |
| Harmful | 36.4(30-43.4; 211) | |  |
| Neither helpful nor harmful | 32.5(25.9-39.7; 188) | |  |
| Helpful | 6(1.1-21; 35) | |  |
| Extremely helpful | 2.4(0.1-30; 14) | |  |
|  |  |  |  |
| Have you ever had a post censored or removed because of visible healed self-harm scars? | | | |
| Yes | 21.1(18.7-23.8; 1047) | | |
| No | 78.9(77.5-80.1; 3908) | | |
|  |  |  |  |
| If yes, do you think having this post censored/removed was helpful or harmful to you at the time? | | | |
| Extremely harmful | 58.3(54.3-62.3; 599) | |  |
| Harmful | 31.3(26.3-36.7; 321) | |  |
| Neither helpful nor harmful | 9.7(5-17.7; 100) | |  |
| Helpful | 0.4(2.2-60.7; 4) | |  |
| Extremely helpful | 0.3(3-69.2; 3) | |  |
|  |  |  |  |
| Have you ever had a post censored or removed that was related to recovery, raising awareness or positive mental health? | | | |
| Yes | 21.1(18.7-23.7; 1044) | | |
| No | 78.9(77.6-80.2; 3908) | | |
|  |  |  |  |
| If yes, do you think having this post censored/removed was helpful or harmful to you at the time? | | | |
| Extremely harmful | 51.1(46.8-55.4; 528) | |  |
| Harmful | 35.8(31-41; 370) | |  |
| Neither helpful nor harmful | 10.6(5.7-18.3; 109) | |  |
| Helpful | 1.6(0.1-25.2; 17) | |  |
| Extremely helpful | 0.9(0.7-38.2; 9) | |  |
|  |  |  |  |
| Would you like the option to be able to keep a post for yourself and just have it removed from public view (as opposed to the post being removed completely)? | | | |
| Yes | 84.6(83.5-85.7; 4191) | | |
| No | 15.4(12.9-18.2; 760) | |  |
|  |  |  |  |
| In 2019 social media platforms increased restrictions on posting and searching for self-harm related content. Did this change what you saw on social media? | | | |
| Yes | 34.9(32.6-37.2; 1736) | | |
| No | 65.1(63.5-66.8; 3245) | | |
|  |  |  |  |
| Did this change the kinds of things you posted on social media? | |  |  |
| Yes | 9.9(7.5-13; 492) | |  |
| No | 90.1(89.2-91; 4479) | |  |
|  |  |  |  |
| Do you think these restrictions help to make social media safer? | |  |  |
| Yes | 49.8(47.8-51.8; 2476) | | |
| No | 50.2(48.2-52.2; 2494) | | |
|  |  |  |  |
| Do you think these restrictions could make someone feel bad or isolated? | | |  |
| Yes | 79.4(78.1-80.7; 3947) | | |
| No | 20.6(18.2-23.2; 1022) | | |
|  |  |  |  |
| Have you ever had a social media account suspended for breaking the rules around self-harm content? | | | |
| Yes | 11(8.5-14; 525) | |  |
| No | 89(88.1-90; 4264) | |  |
|  |  |  |  |
| Did you receive clear messaging from the platform about why your account had been suspended? | | | |
| Yes | 34.2(27.4-41.7; 179) | |  |
| No | 65.8(60.5-70.7; 344) | |  |
|  |  |  |  |
| Do you think having your account suspended was helpful or harmful to you at the time? | | | |
| Extremely harmful | 31.8(24.9-39.5; 166) | |  |
| Harmful | 44.6(38.2-51.3; 233) | |  |
| Neither helpful nor harmful | 14.9(8.2-25.2; 78) | |  |
| Helpful | 6.3(1.2-22; 33) | |  |
| Extremely helpful | 2.3(0.1-33.1; 12) | |  |
|  |  |  |  |
| Have you seen messages like this on social media platforms before? | | |  |
| Yes | 77.9(76.5-79.2; 3619) | | |
| No | 22.1(19.6-24.8; 1027) | | |
|  |  |  |  |
| Would this sort of message stop you from searching from suicide or self-harm content? | | | |
| Yes | 20.8(18.3-23.5; 962) | |  |
| No | 79.2(77.9-80.5; 3665) | | |
|  |  |  |  |
| How likely would you be to try one of the 'tips and support' options such as talking to a friend or distraction activities? | | | |
| Very likely | 2.3(0.5-7.9; 107) | |  |
| Likely | 17.1(14.5-19.9; 792) | |  |
| Neither likely Nor unlikely | 18.6(16.1-21.4; 863) | |  |
| Unlikely | 29.6(27.2-32.1; 1376) | | |
| Very unlikely | 32.4(30.1-34.9; 1507) | | |
|  |  |  |  |
| Have you ever contacted one of the professional sources of help? | |  |  |
| Yes | 34(31.7-36.4; 1577) | |  |
| No | 66(64.3-67.7; 3061) | |  |
|  |  |  |  |
| Do you think these sources of help are relevant to you? |  |  |  |
| Yes | 47.2(45.1-49.3; 2179) | | |
| No | 52.8(50.8-54.8; 2435) | | |
|  |  |  |  |
| Would any of the following make you more likely to seek/accept help? (Tick all that apply) | | | |
| Sources of help specific to your area | 56.7(54.7-58.7; 2362) | | |
| Option to message/text someone | 79.8(78.4-81.2; 3327) | | |
| Option to have someone contact you | 46(43.8-48.3; 1917) | |  |
|  |  |  |  |
|  |  |  |  |
| Would you like the option to add the details of a trusted person to your account who could be contacted if someone were worried about you? | | | |
| Yes | 38.8(36.6-41.1; 1791) | | |
| No | 61.2(59.3-63; 2822) | |  |
|  |  |  |  |
| Would you like more control over the content you view on social media? | 0(0-0; ) |  |  |
| Yes | 87.8(86.7-88.8; 3760) | | |
| No | 12.2(9.6-15.4; 521) | |  |
|  |  |  |  |
| Which of these features would have a positive impact on content? Tick all that apply | | | |
| Easy to mute certain keywords/hashtags | 83(81.7-84.2; 3559) | |  |
| Feature to turn suggested content on and off easily | 65(63.2-66.8; 2788) | |  |
| Changes to settings so that you must switch on content algorithms for key words such as mental health and self-harm | 64.5(62.7-66.3; 2767) | | |
| Guidance on how to control what you see online | 55.1(53-57.1; 2361) | |  |
| Features for people to generate their own specific trigger warnings | 83.3(82-84.5; 3571) | |  |
| Intervention at point of post, this could be letting someone know their post will be censored or asking them to provide a trigger warning for others | 59.9(57.9-61.8; 2566) | | |
| More mental health awareness information | 60.5(58.6-62.4; 2594) | | |
| Space for stories of recovery and positive messages | 54(51.9-56; 2315) | |  |
| Increased moderation | 91(90-91.8; 3900) | |  |
| Changes to suggested content and “for you” pages | 78.1(76.7-79.5; 3350) | | |
| Changes to targeted advertising | 85.4(84.2-86.5; 3661) | | |
| Refinement of censoring/blocking of posts. For example, different policies for healed self-harm scars compared with explicit images | 52(49.9-54.1; 2228) | |  |
|  |  |  |  |
|  |  |  |  |
| Have you ever reported a post because of self-harm/suicide content? | | |  |
| Yes | 58.6(56.7-60.4; 2663) | | |
| No | 41.4(39.2-43.7; 1885) | | |
|  |  |  |  |
| Does this result in the post being removed by platforms? | |  |  |
| No | 40.3(37.4-43.3; 1070) | | |
| Sometimes | 54.1(51.5-56.7; 1437) | | |
| Yes | 5.6(2.6-10.9; 148) | |  |
|  |  |  |  |
| Here is an example of the messaging Facebook shows someone when a friend reports their post for self-harm or suicide content; Have you seen this sort of message before? | | | |
| Yes | 19.7(17.2-22.5; 896) | |  |
| No | 80.3(78.9-81.5; 3647) | | |
|  |  |  |  |
| Would you be more or less likely to seek help after seeing this message? | | |  |
| Much less likely | 8.4(5.9-11.8; 378) | |  |
| Less likely | 10.7(8.2-13.9; 480) | |  |
| Unsure/no difference | 52.8(50.8-54.8; 2365) | | |
| More likely | 24(21.5-26.7; 1075) | |  |
| Much more likely | 4(1.8-8.3; 181) | |  |
|  |  |  |  |
| Do you think this kind of message is helpful? |  |  |  |
| Yes | 66.3(64.6-68; 2981) | |  |
| No | 33.7(31.3-36.1; 1512) | | |
|  |  |  |  |
| Do you think this sort of message could make someone feel isolated or lonely? | | | |
| Yes | 50.6(48.5-52.7; 2277) | | |
| No | 49.4(47.3-51.5; 2222) | | |
