# Supplementary material for: Experiences of Social Media Platforms’ Policies and Restrictions Related to Self-Harm Content: Mixed Methods Study
Source: J Med Internet Res. 2026 Aug 6;28:e73343. doi: 10.2196/73343 (PMC13446788; doi:10.2196/73343)
Supplement: Supplementary file 4 [file jmir-v28-e73343-s003.docx]

# Summary of item nonresponse

|  |  |  |  |  | Gender |  |  | Age |  |  | History of self-harm | | |
| --- | --- | --- | --- | --- | --- | --- | --- | --- | --- | --- | --- | --- | --- |
|  | | | | % non response | chi2 | df | P | chi2 | df | P | chi2 | df | P |
| How old were you when you first saw self-harm or suicide related content on social media? | | | | 0.1 | 2.927a | 2 | 0.231 | 12.382a | 6 | 0.054 | 0 | 2 | 0.184 |
| If yes on which platform (tick all that apply) | | | | 0.1 | 2.309a | 2 | 0.315 | 1.409a | 6 | 0.965 | .308a | 2 | 0.857 |
| Have you ever had more than one social media account and kept one or more just for mental health/self-harm content? | | | | 0.4 | 4.763a | 2 | 0.092 | 4.177a | 6 | 0.653 | 17.940a | 2 | <.001 |
| Have you ever viewed self-harm or suicide content on social media that you did not search for, for example on the “explore” or “for you” pages? | | | | 0.3 | 9.747a | 2 | 0.008 | 3.333a | 6 | 0.766 | 29.072a | 2 | <.001 |
| If yes on which platform (tick all that apply) | | | | 17 | 5.204a | 2 | 0.074 | 2.901a | 6 | 0.821 | 37.779a | 2 | <.001 |
| When I see online content about self-harm this is: [accidental, intentional etc] | | | | 0.2 | 5.204a | 2 | 0.074 | 2.901a | 6 | 0.821 | 37.779a | 2 | <.001 |
| Usually, seeing, sharing or posting online content about self-harm [mood] | | | | 0.2 | 6.034a | 2 | 0.049 | 146.777a | 6 | <.001 | 5.135a | 2 | 0.077 |
| Usually, seeing, sharing or posting online content about self-harm [behaviour] | | | | 0.3 | .210a | 2 | 0.9 | 121.073a | 6 | <.001 | 33.921a | 2 | <.001 |
| As a consequence of seeing self-harm content online, have you ever performed the same (or very similar) self-harm? | | | | 0.4 | 2.501a | 2 | 0.286 | 24.980a | 6 | <.001 | 14.630a | 2 | <.001 |
| As a consequence of seeing self-harm content online, have you ever self-harmed more or more severely? | | | | 0.4 | 1.534a | 2 | 0.464 | 25.583a | 6 | <.001 | 27.400a | 2 | <.001 |
| Have you ever seen a post that has been censored, blurred or marked with a warning such as this? | | | | 5.6 | 9.300a | 2 | 0.01 | 20.237a | 6 | 0.003 | 24.457a | 2 | <.001 |
| Do you think this can draw more attention to the person who posted it? | | | | 5.7 | 8.172a | 2 | 0.017 | 21.733a | 6 | 0.001 | 23.278a | 2 | <.001 |

| How likely are you to click on a post that has been censored in this way? | 5.6 | 8.523a | 2 | 0.014 | 20.163a | 6 | 0.003 | 24.457a | 2 | <.001 |
| --- | --- | --- | --- | --- | --- | --- | --- | --- | --- | --- |
| If this post had a self-harm specific trigger warning/message would this make you more or less likely to click on it? | 5.6 | 9.904a | 2 | 0.007 | 21.485a | 6 | 0.002 | 24.659a | 2 | <.001 |
| Have you ever seen a censored post on an 'explore' or 'for you page'? | 5.7 | 9.312a | 2 | 0.01 | 21.535a | 6 | 0.001 | 23.665a | 2 | <.001 |
| Have you ever had any of your own posts censored or removed by a social media platform because of self-harm content? | 5.7 | 8.855a | 2 | 0.012 | 21.020a | 6 | 0.002 | 26.496a | 2 | <.001 |
| If yes, how long after posting did this happen | 9.1 | 11.890a | 2 | 0.003 | 37.166a | 6 | <.001 | 29.041a | 2 | <.001 |
| If yes, do you think having this post censored/removed was helpful or harmful to you at the time? | 7.3 | 18.151a | 2 | <.001 | 29.648a | 6 | <.001 | 33.999a | 2 | <.001 |
| Have you ever had a post censored or removed because of visible healed self-harm scars? | 6.4 | 8.465a | 2 | 0.015 | 20.867a | 6 | 0.002 | 28.636a | 2 | <.001 |
| If yes, do you think having this post censored/removed was helpful or harmful to you at the time? | 6.6 | 17.452a | 2 | <.001 | 23.596a | 6 | <.001 | 28.846a | 2 | <.001 |
| Have you ever had a post censored or removed that was related to recovery, raising awareness or positive mental health? | 6.5 | 9.770a | 2 | 0.008 | 21.346a | 6 | 0.002 | 37.780a | 2 | <.001 |
| If yes, do you think having this post censored/removed was helpful or harmful to you at the time? | 6.5 | 20.619a | 2 | <.001 | 20.130a | 6 | 0.003 | 24.711a | 2 | <.001 |
| Would you like the option to be able to keep a post for yourself and just have it removed from public view (as opposed to the post being removed completely)? | 6.5 | 11.662a | 2 | 0.003 | 21.104a | 6 | 0.002 | 30.892a | 2 | <.001 |
| In 2019 social media platforms increased restrictions on posting and searching for self-harm related content. Did this change what you saw on social media? | 5.9 | 10.209a | 2 | 0.006 | 21.339a | 6 | 0.002 | 31.200a | 2 | <.001 |
| Did this change the kinds of things you posted on social media? | 6.1 | 8.866 | 2 | 0.012 | 28.9 | 6 | <.001 | 21.683 | 2 | <.001 |
| Do you think these restrictions help to make social media safer? | 6.1 | 10.705 | 2 | 0.005 | 25.337 | 6 | <.001 | 18.968 | 2 | <.001 |
| Do you think these restrictions could make someone feel bad or isolated? | 6.1 | 11.81 | 2 | 0.003 | 30.758 | 6 | <.001 | 20.87 | 2 | <.001 |
| Have you ever had a social media account suspended for breaking the rules around self-harm content? | 9.5 | 18.552 | 2 | <.001 | 38.472 | 6 | <.001 | 23.737 | 2 | <.001 |
| Did you receive clear messaging from the platform about why your account had been suspended? | 20.8 | 11.999 | 2 | 0.002 | 58.422 | 6 | <.001 | 9.563 | 2 | 0.008 |
| Do you think having your account suspended was helpful or harmful to you at the time? | 31.9 | 12.552 | 2 | 0.002 | 42.305 | 6 | <.001 | 9.954 | 2 | 0.007 |
| Have you seen messages like this on social media platforms before? | 12.2 | 29.583 | 2 | <.001 | 36.464 | 6 | <.001 | 29.075 | 2 | <.001 |
| Would this sort of message stop you from searching from suicide or self-harm content? | 12.6 | 32.535 | 2 | <.001 | 37.652 | 6 | <.001 | 38.398 | 2 | <.001 |
| How likely would you be to try one of the 'tips and support' options such as talking to a friend or distraction activities? | 12.3 | 30.392 | 2 | <.001 | 38.652 | 6 | <.001 | 32.7 | 2 | <.001 |
| Have you ever contacted one of the professional sources of help? | 12.4 | 30.313 | 2 | <.001 | 35.761 | 6 | <.001 | 33.776 | 2 | <.001 |
| Do you think these sources of help are relevant to you? | 12.8 | 32.792 | 2 | <.001 | 30.67 | 6 | <.001 | 34.732 | 2 | <.001 |
| Would any of the following make you more likely to seek/accept help? (Tick all that apply) | 21.3 | 5.582 | 2 | 0.061 | 26.573 | 6 | <.001 | 27.346 | 2 | <.001 |
| Would you like the option to add the details of a trusted person to your account who could be contacted if someone were worried about you? | 12.9 | 33.601 | 2 | <.001 | 42.49 | 6 | <.001 | 42.862 | 2 | <.001 |
| Have you ever reported a post because of self-harm/suicide content? | 14.1 | 35.706 | 2 | <.001 | 54.547 | 6 | <.001 | 33.589 | 2 | <.001 |
| Does this result in the post being removed by platforms? | 24.9 | 22.167 | 2 | <.001 | 51.395 | 6 | <.001 | 28.9 | 2 | <.001 |
| Here is an example of the messaging Facebook shows someone when a friend reports their post for self-harm or suicide content. Have you seen this sort of message before? Facebook | 14.2 | 34.413 | 2 | <.001 | 54.339 | 6 | <.001 | 34.859 | 2 | <.001 |
| Would you be more or less likely to seek help after seeing this message? | 15.4 | 44.26 | 2 | <.001 | 46.181 | 6 | <.001 | 36.818 | 2 | <.001 |
| Do you think this kind of message is helpful? | 15.1 | 39.298 | 2 | <.001 | 49.522 | 6 | <.001 | 38.469 | 2 | <.001 |
| Do you think this sort of message could make someone feel isolated or lonely? | 15 | 39.002 | 2 | <.001 | 53.441 | 6 | <.001 | 37.248 | 2 | <.001 |
| Would you like more control over the content you view on social media? | 19.1 | 40.186 | 2 | <.001 | 80.017 | 6 | <.001 | 32.42 | 2 | <.001 |
| Which of these features would have a positive impact on content? Tick all that apply | 20.5 | 44.174 | 2 | <.001 | 64.855 | 6 | <.001 | 34.41 | 2 | <.001 |
| FeatSafe_Response | 19.8 | 45.297 | 2 | <.001 | 76.832 | 6 | <.001 | 40.022 | 2 | <.001 |
