# Supplementary material for: Experiences of Social Media Platforms’ Policies and Restrictions Related to Self-Harm Content: Mixed Methods Study
Source: J Med Internet Res. 2026 Aug 6;28:e73343. doi: 10.2196/73343 (PMC13446788; doi:10.2196/73343)
Supplement: Supplementary file 5 [file jmir-v28-e73343-s004.docx]

# Themes, subthemes, and further examples identified from analysis of in-depth interviews

| Theme | Subtheme | Example |
| --- | --- | --- |
| Experiences of viewing self-harm/suicide related content | Nature of self-harm content e.g. type of content viewed, most popular platforms and whether self-harm content was specifically searched or whether this appeared in suggested content such as ‘newsfeeds’ and ‘for you’ pages | *‘For Instagram, it would be something that would appear on my ‘explore’ page.*  *For Twitter, if would be a recommended tweet on my newsfeed (probably based on the fact that I do follow a lot of mental health / psychology related accounts).*  *For Reddit, that was something I used to actively search for when I was experiencing an eating disorder*  *I guess it really depends on the platform since the algorithm likely works differently on each one’* **Interview** **Participant 1**  *‘****Researcher****: You mention there being a lot of pictures on Instagram, was this something you searched for or did it appear on your newsfeed?*  ***Participant****: At the time I was searching for things to help me express my feelings, I was looking for a set of words to describe - I initially found it under the “depressed” as I wouldn’t have really known what self harm was.*  *It then became something I was exposed to irregularly because I was considering doing it*  ***Researcher****: Did you find it appeared in your suggested content once you'd searched for it?*  ***Participant****: Yes that was the case’* **Interview Participant 15**  ***Researcher****: And were you searching for self-harm or did this just appear on your newsfeed?*  ***Participant****:: It just came up [..] I started following vent accounts and stuff and then saw more as time went on*  ***Researcher****: So you saw more once you started following certain accounts?*  ***Participant****: Yeah and then more explicit accounts would be recommended’* **Interview Participant 16** |
|  | Age and safety of younger people | *‘ I think I first starting seeing self harm on Instagram when I first got it, so when I was 12. Explicit photos of people's self harm injuries mostly.’* ***Interview Participant 2***  *“I would say recently it’s become more regulated but when I was younger there was an influx of pictures under the hashtag #selfharm on Instagram, it had pictures of self-harm with directions on how to do it. I remember that quite well - I’ve recently seen content on twitter with self-harm ideation but that’s all. I try and limit my interaction […]* *I was 11 - understandably under the age of Instagram joining age however there wasn’t a lot to protect children from joining”* **Interview Participant 15** |
|  | Effect of self-harm content on mood or behaviour | *“Seeing the more general self-harm content (e.g., physical self-harm as opposed to eating behaviours) would also exacerbate my negative feelings and make me feel more unhappy and like I should engage in similar behaviours […] It’s unrelated to mood / feelings, but I do also think that accessing written descriptions of self-harm also gave me more ‘ideas’ of ‘different’ ways to self-harm that I personally wouldn’t have thought of if I never came across that content in the first place.”* **Interview Participant 1**  *‘I think it had an impact on how I perceived self-harm, in the sense that if they were smaller cuts or didn’t bleed etc, then it didn’t really “count” or wasn’t severe - now that I’m older, I have a very different view, but I can understand why at 13/14 I was consuming content that would lead me to think that am way’* **Interview Participant 6** |
| Online communities | Importance of community support | *‘Yes urging you to seek support because it’s not helpful as a) there’s no support or b) you don’t want to’* **Interview Participant 5**  *‘****Participant****…especially alternatives to cahms because there’s so much information online-especially on tiktok of people describing their cahms experience-that puts people off 100%*  ***Researcher****: Could you tell me a little more about that and how they put people off?*  ***Participant****: Well I guess it depends on what you would be going to cahms for because I’ve seen on tiktok people saying that cahms for ED stuff is great but for anxiety/depression and other mental health disorders, a lot of these peoples experiences are quite negative. With cahms workers saying stuff like, go have a cuppa, go a walk, socialise more, or basically tries to suggest that your mental health is your own fault […] With my personal experience with cahms is also not that great, so I would understand when younger people potentially going through something would see this content being posted and be put off with getting help’* **Interview Participant 7**  *‘It’s strange because even though there is harmful content on it, I find a lot of enjoyment and entertainment out of it! I think it can be a helpful/community space is used correctly, I’ve made a lot of friends over social media ☺️*’ **Interview Participant 7** |
|  | Dedicated recovery accounts | *‘I think in the past I initially followed different people work mental health recovery accounts (ones where they share their personal story, progress, photos of themselves, mental health accomplishments etc.) as ‘inspiration’ or to provide a sense of ‘hope’ that I could get my own mental health back on track.*  *For awhile it was helpful, but after some time I would find myself comparing myself to the people on those different accounts and thinking that my own mental health recovery was somehow flawed or didn’t look as good as theirs*  *So I guess I initially found them to be positive, but over time of just fuelled comparison and interfered with my own mental health recovery. Which is what led to me unfollowing them.*  *I know that these accounts can be really helpful and encouraging for a lot of other people though! So I think it really just comes down to individual mindset and circumstances.’* **Interview Participant 1**  *‘I'd like to see more things encouraging recovery, and less posts normalising having a bad mental health and not trying to recover. I see a lot of people who don't want to even try to get better, and it's really discouraging.’* **Interview Participant 2**  *‘****Researcher****: Did you ever have a dedicated mental health or recovery account?*  ***Participant****: Yes I did on both a forum then Tumblr and Instagram*  ***Researcher****: Were these completely anonymous and separate from your personal accounts?*  ***Participant****: Yes absolutely! No one irl [in the real world] knew about them*  ***Researcher****: Did you find this helpful?*  ***Participant****: That’s a difficult question because the answer is yes and no. Helpful to feel less alone but kept me caught up with mental illness as my identity and getting worse as it sparked a competitive edge in me’* **Interview Participant 5** |
| Experiences of self-harm/suicide content restrictions | Restrictions effective/necessary | *‘****Participant****: Although it’s a good thing, I remember feeling frustrated at the time*  ***Researcher****: could you tell me a bit more about that experience?*  ***Participant****: I wanted to view self harm content to feel less alone and it was frustrating I couldn’t because sometimes you want to trigger yourself or see something you relate to’* **Interview Participant 5**  *‘At that time, none of those sorts of accounts/ pages were private or restricted, whereas now I’m almost certain it would be harder to access, or warnings would be given beforehand etc’* **Interview Participant 6**  *‘Definitely yeah, It means that no one could ever stumble across it by accident or become upset/triggered by something they didn’t intend on seeing, and I think as well it will have helped a lot in terms of self harm not being seen as “trendy”. It’s quite hard to describe, because it was never “cool” or anything, but I think for some people it was almost like an expectation if you were feeling a certain way, that’s what you would do. Having it under tighter wraps means its not advocated as such, it’s less encouraging or harder for young people especially to be influenced in that way (if that makes sense*)’ **Interview Participant 6**    *‘In general, when I was younger access to content displaying self-harm was more prominent but with the trigger/content warnings on things now it’s a little more regulated. The sort of content being-what pills to take that will kill you, videos of people cutting themselves (this was mainly on YouTube early 2010-15) from what I remember and I’m sure there was more out there Twitter still is particularly bad for pro-anorexia/food self-harm [..] I remember seeing stuff like this from the ages of say 9-15’* **Interview Participant 7** |
|  | Restrictions ineffective | *‘I think it become worse to be honest. It’s easier to find although it does come with a warning now if you search for self harm’* **Interview Participant 3**  *‘There’s more control than there used to be. But not fully’* **Interview Participant 16** |
|  | Restrictions making someone feel bad or isolated | *“I think so, especially on YouTube and similar sites with super restrictive content guidelines, it can make it hard for people to talk about their past experiences”* **Interview Participant 2**  *“Stopping all discussion may be counter intuitive and remove life lines for some people”* **Interview Participant 14** |
| Censoring, blurring or removing content | Role of content/trigger warnings and whether this is an effective barrier against harmful content | *‘If someone is in a mindset where they already feel horrible about themselves and their mood is very low and they have a low sense of self, they may be in a mindset where they’re actively trying to trigger or harm themselves by accessing this type of content. In this case, trigger warnings wouldn’t be very helpful because the person would want to view that post regardless.’* **Interview participant 1**  *‘I have seen that sensoring yeah, a lot during the BLM movement around George Floyd, for example. I think it depends on the account whether or not I would click on it - some that are for things like BLM, Ukraine, the Palestinian conflicts etc, they are sensored but I often want to know the information and be up to date where I can. For accounts that lean more towards like “memes” or entertainment, I probably skip past them, just because they don’t interest me and it’s almost like the cost benefit analysis I guess, haha, it just doesn’t hold relevance to me’* **Interview Participant 6**  *‘I think on Twitter a lot of individuals who discuss content will state, “TW/“ and then whatever it may be, so it allows the individual to make a choice. It could definitely be beneficial because not everything will provoke the same responses in people, and I have found that sometimes if I proceed to see a post im like “why did they sensor this” etc’* **Interview Participant 6**  *‘Often, the content is blurred/censored but the reason is unclear. I think content warnings can be more specific and alternative descriptions could be provided.’* **Interview Participant 10** |
|  | Experiences of having own posts blurred, censored or removed. This included posts being removed for self-harm content, visible healed scars, mental health and recovery | *‘I can’t recall ever having my own content reviewed no, I hear the most about that happening on TikTok - I doubt it’s relevant to self harm in the same way, but I know I follow a young girl who shares her Tourette’s journey and often has her videos removed because of her tics/ if she is swearing etc. it can cause problems for her because she is advocating awareness and education for Tourette’s, which there is a lot of misconception of, but it’s misunderstood by guidelines.’* **Interview Participant 6** |
| Reporting of posts | Procedure when a post is reported | ***‘****Instagram often send a message saying they are too busy to review all posts which I don’t find helpful at all’* **Interview Participant 3**  ***‘….****and for social media companies to actually investigate those reports, because it seems that even when you report that, the post or account stays up’* **Interview Participant 4**  *‘That’s the exact thing which frustrates me about Instagram because it will show me less posts but generally the triggering content isn’t removed :((‘* **Interview Participant 7**  *‘To make social media safer, I think reports under categories such as self harm should be prioritized’* **Interview Participant 8** |
|  | Reaching out to someone as alternative to reporting posts | *‘I very regularly reach out, it’s very regular that actual self-harm is done by an “anonymous” user, so you don’t know whose actually behind the profile so it’s difficult to contact anyone. If I was concerned about suicide or ideation, I would likely contact the police for a wellness check and inform them why I was concerned’* **Interview Participant 15** |
| Help messaging and signposting | Help messaging and help seeking | *‘I’ve followed accounts in the past where they would be reported on Instagram where they get the “we’re reaching out to offer help” message.*  *I think a ‘get support’ button may have flashed up before but I’ve never actually clicked on it’* **Interview Participant 1**  ***‘Researcher****: Would you be likely to contact a source of support if you saw this kind of message?*  ***Participant:*** *Oh yes definitely!’* **Interview Participant 3**  *‘I am actually quite a likely person because I want help that is accessible to me-that isn’t just cahms or going through the NHS-I know of SHOUT which I have used before which is really helpful’* **Interview Participant 7**  *‘If I wanted support it would be good. If I didn’t it would feel controlling. But usually I want to feel better and this could help’* **Interview Participant 14** |
|  | Nature of messaging | *‘I like that there are multiple options, it feels more accessible than just “this is one website we recommend” because that doesn’t suit everyone, this is more encouraging’* **Interview Participant 6**  ***‘****I’ve seen ones on Instagram-I’ve seen ads on tiktok saying resources where you can gain access to therapists online/phone numbers but those are usually American so it doesn’t really matter to people in the UK’* **Interview Participant 7** |
| Possible changes to platform features | Age restrictions and verification | *‘…things like proving your age like YouTube has done-I couldn’t watch a rhianna music video until I turned 18 due to the age restrictions haha-you had to verify with a bank account or something I think. So maybe better verification like that if harmful content is gonna possibly be available […] I believe if YouTube had this when I was younger then a lot of the clips, I saw of people harming themselves I wouldn’t have seen-e.g., the [name] suicide video was one that still sticks in my mind. I guess it doesn’t stop harmful content altogether, but it makes it harder for people to view. I think the regulations that are coming into play now like YouTube regulations of age and blockage of certain videos as well as Instagram’s Tw/Cw [trigger warning/content warning] is definitely helpful. Of course, school gives you lessons on how to be safe online, but they don’t really do much when you just happen to stumble on things that are so graphic/triggering.’* **Interview Participant 7** |
|  | More control over suggested content such as newsfeeds, ‘explore’ and ‘for you’ pages. This includes the need for features to be intuitive, user friendly and easy to fine | ***‘…****for younger people, it might be better for it to be automatically not suggested’* **Interview Participant 4**  *‘I can’t seem to find a feature to make it not appear on my for you page’* **Interview Participant 11**  *‘When viewing suicidal content I've often deleted my history afterwards in an effort to avoid it being suggested at a later date’* ***Interview Participant 13***  *‘I would say to make the verification and joining process a little trickier, as you only need to input a birthday and you have access. I would suggest special attention paid to limit content shown on an under 18’s account & to understand who is behind the accounts being made, I would suggest a photo verification or ID’* ***Interview Participant 15***  *‘I think they should a switch to turn of possible triggering content’* **Interview Participant 17** |
|  | More positive and uplifting content/content to raise awareness | *‘……I think that instead of suggesting accounts similar to what the person is seeking out, they could instead recommend accounts focusing on recovery and accepting the reality of self-harm, depression etc without romanticising it or allowing it to be unmonitored’* **Interview Participant 4** |
|  | Guidance for posters/restrictions when creating a post – this included guidance on how to post safely including prompts when posting new content | *‘It would be useful if post had to be tagged with any major warnings, maybe out of a list, when posted. So instead of just being able to block posts explicitly tagging self-harm, you could block any post where it's flagged as a warning.*’ **Interview Participant 2**  *‘I think a lot of people like TikTok for sharing their experiences/ relating to others who have the same, which is extremely different from where it started with musically, which I don’t think is a problem. I think they could do well from encouraging people to use the appropriate #s, and then filtering say “#selfharm” for those who search for it, like Instagram with appropriate warnings etc, so no one randomly can just see it without wanting to etc’* **Interview Participant 6** |
|  | Improvements to signposting | *‘…instant support would be better like a message support’* **Interview Participant 5**  *‘I think it would be better to send an automatic response to the messaging part of apps as well. I might look at it but if I wasn't in a good place I don't know if I would’* **Interview Participant 12** |
