# Supplementary material for: Experiences of Social Media Platforms’ Policies and Restrictions Related to Self-Harm Content: Mixed Methods Study
Source: J Med Internet Res. 2026 Aug 6;28:e73343. doi: 10.2196/73343 (PMC13446788; doi:10.2196/73343)
Supplement: Supplementary file 6 [file jmir-v28-e73343-s005.docx]

# Integration of survey and interview data

| **Theme** | **Survey Data** | **Interview insights** |
| --- | --- | --- |
| Experiences of viewing self-harm/suicide related content | 43.2%(41.2-45.3; 1405/5283) of respondents reported viewing self-harm content either always or often accidental  82.9%[81.7-84.0; 4376/5280] had viewed self-harm content that they did not search for  68.8%(68.3-70.3; 3639/5290) of survey respondents were aged 11-14, 17.4%(16.5-20.1; 921/5290) aged 10 years or under and the first time they viewed self-harm content on social media.  34.9%(32.7-37.1; 1844/5282) of survey respondents reported a worsening of mood as a result of seeing or sharing self-harm content online  76.8%[75.4-78.1; 3806/5021]) reported self-harming more severely as a result of seeing self-harm content online | Self-harm/suicide content often driven by algorithms on ‘explore’ or ‘for you’ pages, often at young ages  This content can provide information on new methods and shapes perceptions of severity  Impact on mood or behaviour said to vary over time and is dependent on emotional state |
| Online communities | 39.2%[37.1-41.3; 2065/5273]) of respondents had maintained an account which is separate from their personal account dedicated to mental health or self-harm recovery. | Communities reduce isolation and facilitate connection with others  Mixed narratives of recovery accounts and whether these reduce isolation or lead to comparison with others and identity reinforcement centred around mental health and self-harm |
| Experiences of self-harm/suicide content restrictions | 34.9%[32.6-37.2; 1736/4981] reported that the 2019 change in policy changed what they saw on social media and just 9.9%(7.5-13.0; 492/4479) stated that this changed what they posted.  49.8%(47.8-51.8; 2476/4970) felt that these restrictions made social media safer and 50.2%(48.2-52.2; 2494/4970) felt that they did not  79.4%[78.1-80.7; 3947/4969]) of survey respondents felt that restrictions could make someone feel bad or isolated | Mixed perceptions with self-harm/suicide content said to be both harder and easier to find following additional restrictions  Restrictions may remove support spaces and silence discussion |
| Censoring, blurring or removing content | >90% stated that they would be either likely or very likely to click on a censored post  40% said that they would be more likely or much more likely to view a post with a self-harm specific warning  89.1%[88.2-90.0; 4449/4991]) felt that having a post censored, blurred or marked with a warning could draw more attention to the person who posted it  12.2% (9.8-15.1; 609/4992) of survey participants had previously had a post censored or removed because of self-harm content, 21.1%(18.7-23.8; 1047/4955) because of visible self-harm scars and 21.1%(18.7-23.7; 1044/4952) had a post censored or removed that was related to recovery, raising awareness or positive mental health  Having posts censored or removed for self-harm, visible scars or positive mental health was experienced as either harmful or extremely harmful to participants at the time (Table 3). This was particularly the case for individuals who had posts censored or removed due to visible healed self-harm scars where almost 90% of participants experienced this as either harmful or extremely harmful to them at the time | Self-harm specific warnings allow users to make an informed choice in whether to view content. Generic warnings said to be a source of curiosity.  Acknowledgement that they may continue to actively seek and view content that they know if harmful to them  Frustration with inappropriate censoring of content (e.g. recovery, awareness, scars)  Importance of visibility of recovery-oriented content and potential to feel that stories are being silenced when these are censored or removed |
| Reporting of posts | 58.6%[56.7-60.4; 2663/4548]) of survey respondents had reported a post created by someone else because of self-harm/suicide content. | Frustration that harmful content is not always removed  Individuals report regularly reaching out to individuals they are concerned about rather than reporting posts |
| Help messaging and signposting | 77.9%[76.5-79.2; 3619/4646]) of survey respondents had seen messaging signposting to sources of help  34.0%(31.7-36.4; 1577/4638) had contacted one of the professional sources of help  52.8%[50.8-54.8; 2179/4614] of survey respondents felt that these sources of help are not relevant to them  79.8%[78.4-81.2; 3327/4165] of survey respondents would be more likely to seek/accept help if they could access a messaging or texting service instead of helpline  56.7%(54.7-58.7; 2362/4165) would be more likely to seek/accept help specific to their local area  46.0%(43.8-48.3; 1917/4165) would prefer having to option to have someone contact them | Individuals report seeking help following these messages  Helplines often generic or based in America.  Preference for sources of help specific to the UK/local area and messaging options |
| Possible changes to platform features | 97.8%(86.7-88.8; 3760/4281) of respondents stated that they would like more control over the content they viewed on social media  Further changes show in Figure 1 included increased moderation, changes to targeted advertising and suggested content, refinement which posts are censored/blocked and space for stories of recovery and positive messages | Strong emphasis on control, refinement of censorship, greater visibility of recovery-oriented content, stronger age verification, guidance for posters/content creators and improvements to signposting including support through instant messaging rather than helplines |
